# 3D imaging-driven assembly of multispecies biofilms with antagonistic activity against undesirable bacteria

**DOI:** 10.1093/ismeco/ycaf156

**Published:** 2025-09-05

**Authors:** Virgile Guéneau, Laurent Guillier, Cécile Berdous, Marie-Françoise Noirot-Gros, Guillermo Jiménez, Julia Plateau-Gonthier, Pascale Serror, Mathieu Castex, Romain Briandet

**Affiliations:** Université Paris-Saclay, INRAE, AgroParisTech, Micalis Institute, Jouy-en-Josas 78350, France; Lallemand SAS, Blagnac 31702, France; LabCom Biofilm1Health, Lallemand SAS & Micalis Institute, INRAE, AgroParisTech, Université Paris-Saclay, Jouy-en-Josas 78350, France; French Agency for Food, Environmental and Occupational Health & Safety (ANSES), Laboratory for Food Safety, Salmonella and Listeria Unit, Paris-Est University, 14 rue Pierre et Marie Curie, Maisons-Alfort 94701, France; Université Paris-Saclay, INRAE, AgroParisTech, Micalis Institute, Jouy-en-Josas 78350, France; LabCom Biofilm1Health, Lallemand SAS & Micalis Institute, INRAE, AgroParisTech, Université Paris-Saclay, Jouy-en-Josas 78350, France; Université Paris-Saclay, INRAE, AgroParisTech, Micalis Institute, Jouy-en-Josas 78350, France; LabCom Biofilm1Health, Lallemand SAS & Micalis Institute, INRAE, AgroParisTech, Université Paris-Saclay, Jouy-en-Josas 78350, France; Lallemand SAS, Blagnac 31702, France; Lallemand SAS, Blagnac 31702, France; Université Paris-Saclay, INRAE, AgroParisTech, Micalis Institute, Jouy-en-Josas 78350, France; Lallemand SAS, Blagnac 31702, France; LabCom Biofilm1Health, Lallemand SAS & Micalis Institute, INRAE, AgroParisTech, Université Paris-Saclay, Jouy-en-Josas 78350, France; Université Paris-Saclay, INRAE, AgroParisTech, Micalis Institute, Jouy-en-Josas 78350, France; LabCom Biofilm1Health, Lallemand SAS & Micalis Institute, INRAE, AgroParisTech, Université Paris-Saclay, Jouy-en-Josas 78350, France

**Keywords:** antagonistic biofilms, microbial pathogens, synthetic microbial communities, *Bacillus* spp, *Pediococcus* spp, microbial growth modelling, 4D live-cell imaging

## Abstract

Engineered synthetic microbial communities (SynComs) forming biofilms with antagonistic activity offer a promising strategy in biotechnology to prevent harmful bacterial settlement and reduce reliance on chemical antimicrobials. However, strain selection criteria and antagonistic mechanisms remain unclear. This study presents a bottom-up approach integrating 3D fluorescence imaging with high-throughput analysis of multistrain biofilms. Our findings reveal that competitive strains against undesirable bacteria may also exclude desirable community members, highlighting the need for compatibility control in SynCom assembly. SynComs composed of *Bacillus velezensis* and *Pediococcus* spp. enhanced pathogen exclusion compared to single strains. Temporal analysis of biofilm interactions, supported by mathematical models, showed that pathogen exclusion was primarily driven by nutritional competition (Jameson effect) with additional specific interference dynamics (prey–predator Lotka-Volterra model). Furthermore, pre-established SynComs significantly increased pathogen inhibition, indicating a distinct biofilm-associated exclusion effect. These insights provide a framework for SynCom assembly and refine our understanding of interaction dynamics driving antagonistic biofilm applications.

## Introduction

Microorganisms predominantly exist as biofilms, intricate communities embedded in a matrix, thriving at interfaces with properties distinct from planktonic lifestyles [1–5]. Biofilms typically comprise multiple species engaged in various interactions, including resource as well as interference competition [6, 7]. Deciphering these complex interaction networks to predict biofilm behaviour and functions is a pivotal challenge in microbial ecology.

In line with the ‘One Health’ concept to rationalise the use of chemical antimicrobials, biotechnological solutions are being developed to regulate interactions within complex biofilm communities through guided microbial ecology [8]. For instance, single microbial strains or multistrain consortia, often referred to as synthetic microbial communities (SynComs) [9, 10] capable of forming biofilms with antagonistic activity against undesirable microorganisms can be intentionally introduced in the food chain [11], directly onto hosts [12, 13] but also more recently on surfaces such as livestock buildings [14].

A competitive interaction can be established between bacterial species, where one species dominates while inhibiting the growth or survival of the other, potentially leading to its exclusion [15]. Pathogen exclusion can be attributed to nutritional [16] and spatial competition [17], as well as the secretion of interfering molecules through quorum-sensing modulation [18], bacteriostatic [19], or bactericidal effects [20]. Each exclusion dynamic can be represented using mathematical modelling tools based on temporal analyses [21]. The Lotka-Volterra predator–prey model, originally developed for higher organisms, has been adapted to microbiology to describe systems where the secretion of interfering molecules drives the decline of the ‘prey’ population [22]. Another model that describes the Jameson effect, explained as the competition between species for environmental resources to maximise their growth and population, can also be applied. The Jameson model accounts for a nutritional competition which results in the deceleration of the population growth when the common resource(s) are exhausted [16]. These models provide quantitative parameters describing the evolution of the partners and their mutual influence. However, quantifying antagonism typically relies on assessing planktonic interactions, which do not accurately reflect real conditions under which microbial communities reside in biofilms [23]. Furthermore, the inclusion of multiple strains in SynComs lacks clear justification and assurance of compatibility. In addition, the enhanced effects of SynComs compared to their constituent strains are often unclear.

To improve SynCom design for antagonistic biofilms, we developed a 3D imaging-based pipeline. Strains were selected for their ability to coexist in the same biofilm without excluding partners. Our goal was to identify compatible strains that, together, enhance pathogen exclusion and biofilm formation beyond individual capabilities. This bottom-up approach relies on nondestructive observation of multispecies biofilm phenotypes using high-content screening confocal laser scanning microscopy (HCS-CLSM) combined with genetically engineered fluorescent strains and dedicated image analysis [24]. The collection of tested bacterial isolates comprised 16 *Bacillus*, two *Paenibacillus,* and two *Pediococcus* genus. These genera are renowned for their biofilm-forming abilities [25, 26], exclusion capabilities [20, 27], and versatile applications in biotechnology [11, 28]. Moreover, *Pediococcus* and *Bacillus* genus are commonly found in various environments, including on surfaces in livestock buildings, which are potential targets for antagonistic biofilm applications [14, 29]. All selected strains are suitable for industrial use and are listed on the EFSA QPS list [30]. Finally, the *Paenibacillus* genus was chosen for its specific biofilm-forming properties [31, 32]. The 20 candidate strains were screened for their impact on the growth and establishment of several pathogenic bacteria affecting people and/or animals, including *Staphylococcus aureus*, *Enterococcus cecorum*, *Escherichia coli*, and *Salmonella enterica Serovar Enteritidis* (*S. enterica*), in two submerged mixed-species biofilm models developed for this study.

Through 4D (xyzt) HCS-CLSM imaging of interspecies interactions in biofilms, the underlying types of dynamics driving exclusion of pathogens by the SynComs were modelled. Moreover, our modelling of biofilm interaction curves showed that these dynamics depend on the initial quantities of each partner in the mixed biofilm and are specific to the biofilm lifestyle.

Together, these results allow for the rationalisation of SynCom formulation for antagonistic biofilm application to limit pathogen growth and establishment on surfaces, while understanding the families of interactions involved.

## Materials and methods

### Bacterial strains and genetic constructs

The wild-type (WT) bacterial strains and genetic constructions used in the study are listed in Supplementary Fig. S1. The phylogenetic analysis of the strains was conducted using the bacterial phylogenetic tree service provided by BV-BRC [33]. The phylogenetic tree was then constructed using the default codon tree method. For species assignments, the genome sequences were compared to those of the closest species within the *Bacillus* or *Pediococcus* genus in the BV-BRC database, using 1000 genes with a tolerance for five deletions. Subsequently, the collection of bacterial isolates was ordered based on their phylogenetic distances (Supplementary Fig. S2). The transformation protocol using the pCM11 plasmid derivatives, carrying the GFP or the mCherry-encoding genes, was adapted for each strain. Briefly, WT *E. coli* and *S. enterica* were transformed using a standard heat shock protocol [34]. The protocols for preparing electrocompetent cells and transforming *E. cecorum* were adapted from Dunny *et al.* [35]. *B. velezensis* was transformed based on its natural competence [36] using a methodology inspired by Dergham *et al.* [37]. The plasmid stability assay is presented in Supplementary Fig. S3.

### Biofilm models

All experiments were conducted at 30°C using Tryptic Soy Broth (TSB) medium (BioMérieux, France) supplemented with antibiotics (5 μg/ ml erythromycin for Gram-positive species or 100 μg/ ml ampicillin for Gram-negative species) when appropriate. 5 ml overnight cultures (16–18 h), inoculated from a glycerol stock at −80°C, were centrifuged at 5000 g for 5 min and then re-suspended in fresh TSB medium prior to conducting each experiment. For submerged biofilms, bacteria were cultivated at the bottom of a μClear® 96-well plate (Greiner Bio-one, France), which is compatible with high-resolution fluorescence microscopy.

**Figure 1:**
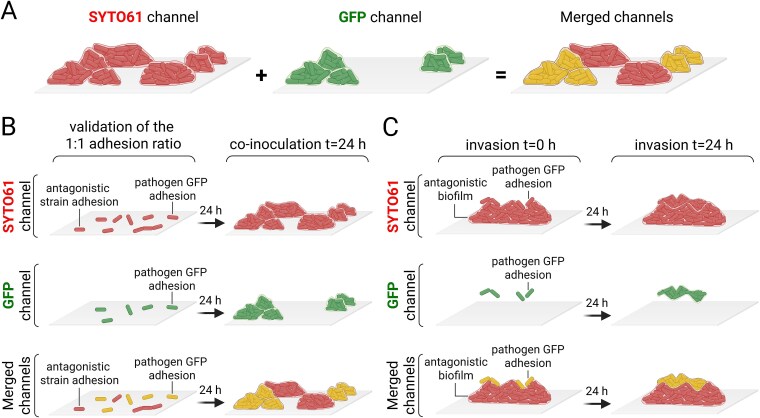
Co-inoculation models used to study interactions in multi-species biofilms by confocal laser scanning microscopy (CLSM). (A) Staining strategy used to distinguish candidate antagonistic bacteria from pathogens. The entire biofilm is stained using SYTO61 to visualise the entire population in red (i.e. pathogens and tested strain candidates with potential antagonistic activity), while the pathogen expresses GFP. Subtracting the green signal from the red signal allows for the estimation of the proportion of antagonistic bacterial candidates. (B) In the co-inoculation model, adhesion ratios of both pathogenic and antagonistic strain candidates were determined by image analysis of CLSM observations. Following adhesion with these predetermined ratios, biofilms were incubated for 24 h before being observed with CLSM. (C) In the invasion model, a planktonic culture of the pathogen was added to a pre-established biofilm for 24 h prior CLSM observation. After adhesion, an incubation of 24 h was followed by CLSM observation.

We developed two co-incubation submerged biofilm assays, which were observed using CLSM (Fig. 1A). One focuses on studying the competition between two types of partners when they are present in equal amounts at the beginning of the experiment (co-inoculation) (Fig. 1B), while the other investigates the potential preventive effect of antagonistic biofilms on the adhesion and proliferation of undesirable bacteria on surfaces (invasion) (Fig. 1C).

Bacterial counts from biofilms were performed by detaching the biofilms from the bottom of 96-well plates through successive pipetting, combined with mechanical scraping: each well bottom was scraped 10 times horizontally and 10 times vertically using a pipette tip (Supplementary Fig. S4). The recovered suspension was transferred to 1.5 ml Eppendorf tubes and vigorously vortexed for 5 s to ensure homogenization. The resulting solutions were then serially diluted in physiological water and plated on Petri dishes containing selective media for enumeration.

For endpoint biofilm visualization, 50 μl of a solution containing SYTO9 (green), SYTO61 (red), or DAPI (blue), three cell-permeable nucleic acid dyes (Invitrogen, Carlsbad, CA, USA) diluted in saline solution (NaCl 9 g/L), was added to each well to achieve a final concentration of 2 μg/ ml. In kinetic measurements, the FM4–64, a lipophilic red dye that binds to cell membranes (Invitrogen, Carlsbad, CA, USA) was used at a vital concentration of 1 μg/ ml [38].

### Co-inoculation model

The initial adhesion biovolume between the fluorescent strain and the nonlabelled strains was standardised using the protocol described by Guéneau *et al.* [24], which is based on a dual labelling by the GFP and the SYTO61 (Supplementary Fig. S5). Three initial adhesion rates were tested in kinetic experiments. The first comprised 10 times more pathogens compared to the antagonistic strain candidate(s) (ratio P > B). The second had the same amount of antagonistic strain candidate(s) compared to the pathogens (ratio P ≈ B). The third comprised 10 times more antagonistic strain candidate(s) compared to pathogens (ratio P < B). For biofilms composed of more than two species, an initial adhesion step was also performed. Culture volumes were adjusted to ensure equal biovolumes of candidate antagonistic bacteria versus pathogens.

In practice, overnight cultures of the GFP-labelled and genetically unlabelled strains were diluted in 1 ml of TSB to achieve the desired adhesion ratio. 200 μl of the bacterial solution were added to the μClear 96-well plates and allowed to adhere statically at 30°C for 1.5 h. The supernatant was then replaced with fresh TSB, and the cultures were incubated for 24 h at 30°C. The same protocol was employed to assess the compatibility between the GFP-labelled *B. velezensis* strains and the collection of tested strain candidates with potential antagonistic activity. Imaging was performed using a Zeiss LSM 700 inverted confocal laser scanning microscope (Carl Zeiss, Germany) equipped with a 405 nm excitation laser for DAPI.

### Invasion model

50 μl of a GFP-labelled pathogen suspension was added to wells containing a 24- h antagonistic biofilm candidate at 30°C for 1.5 h. Subsequently, the supernatants were replaced with 200 μl of fresh TSB. CLSM acquisitions were conducted either directly (invasion t = 0 h) or after 24 h of growth at 30°C (invasion t = 24 h). Before acquiring images, 50 μl of a TSB solution containing SYTO61 or FM4–64 was added to the wells.

### Interaction between macro-colonies

Following homogenisation by rapid vortexing for 5 s, 3 μl of each culture were transferred to a well of a six-well plate containing 4 ml of TSA 1.5% agar. The drops of the two cultures to be tested for interaction were placed next to each other in the same well, with a distance of 1 mm between the edges of the two drops. The samples were dried under a hood for 10 min and incubated at 30°C for 4 days.

### 3D fluorescent imaging

3D fluorescent imaging was conducted using a Leica SP8 AOBS inverted high-content screening confocal laser scanning microscope (HCS-CLSM, LEICA Microsystems, Germany, MIMA2 platform of INRAE, https://doi.org/10.15454/1.5572348210007727E12). Biofilm images were acquired using a 63× water immersion objective with a numerical aperture of 1.2. Imaging was performed at a scan frequency of 600 Hz, with z-stacks acquired at 1 μm intervals to capture the full vertical structure of the biofilm. For each well, imaging fields were randomly selected across the well surface to minimise positional bias and ensure representative sampling. The images had a resolution of 512 × 512 pixels, covering a physical area of 184.52 μm × 184.52 μm with a pixel size of 0.361 μm. For fluorescence detection, SYTO9 and GFP were excited with an argon laser at 488 nm, and the emitted fluorescence was collected using a hybrid detector (HyD LEICA Microsystems, Germany) in the range 500–550 nm. FM4–64 was excited through the use of a helium-neon laser at 561 nm, and the emitted fluorescence subsequently collected with a hybrid detector in the range of 600–750 nm. Excitation of SYTO61 was achieved at 633 nm and the emitted fluorescence was collected with a hybrid detector in the range of 650–750 nm.

For 4D (xyzt) live-cell acquisitions, the temperature was maintained at 30°C and 150 μm stacks were automatically acquired every hour using the high-content screening (HCS) mode of the confocal microscope. Each experiment included between three and six biological replicates, with four technical replicates per biological replicate, thereby providing at least 12 technical values per condition.

### Confocal laser scanning microscopy image analysis

2D projections of biofilms and movies were generated using IMARIS 9.3.1 software (Bitplane, Zurich, Switzerland). The shaded areas of the 2D biofilm images represent XZ projections generated by the IMARIS software. These allow visualisation of the height and overall 3D structure of the biofilm. Quantitative analysis of image stacks were performed using the BiofilmQ software [24, 39]. Each fluorescence channel was analysed separately using the OTSU thresholding method, and ‘global biofilm properties’ were selected to extract the biovolume of the binarised images. Total biovolumes per field of view were normalised to the surface area (μm^3^/μm^2^) to better reflect values at the scale of a single bacterium, thereby allowing direct comparison of biofilm biovolumes across images acquired with different field sizes.

To assess the activity of *Bacillus* species strains against pathogens, the GFP biovolume (μm^3^/μm^2^) of submerged mixed biofilms was quantified and normalised to the biovolume of biofilms of a specific pathogen labelled with GFP. To standardise the measurement of antipathogenic activity, GFP-labelled pathogen biovolume values were rescaled to a range of 0–1 using min-max normalisation:


(1) \begin{align*} {x}_{\mathrm{rescaled}}=\frac{x-{x}_{min}}{x_{max}-{x}_{min}} \end{align*}

where x is the raw biovolume value and x_rescaled_ is the normalised value. The resulting values were then inverted (1 − x_rescaled_) so that a score of one corresponded to the highest exclusion activity and 0 to the lowest. This transformation allowed direct comparison of exclusion efficacy between different bacterial isolates and conditions.

### Modelling microbial growth and competition

In this study, two indicators of competition were modelled, ‘growth potential’ and ‘growth rate’. ‘Growth potential’ is defined as the ability of microbial strains to increase their biovolume within a biofilm under varying environmental and competitive conditions. This parameter is quantified by calculating the increase in biovolume, specifically by subtracting the initial biovolume N_0_ from the final biovolume 𝑁_f_. The modeling approaches described below are employed to quantify both growth rate and growth potential.

GFP-measured biovolumes served as model inputs for both pathogens. For *Bacillus* in monoculture, biovolumes corresponding to the FM4–64 marker were used. In co-culture, antagonistic strain candidate(s) biovolumes were determined by subtracting the GFP-biovolume of the co-inoculated pathogen, from the total biovolume.

The nlsMicrobio [40] and gauseR [41] packages were respectively used to fit the Barnayi&Roberts growth model, Jameson-type and Lotka-Volterra models to biovolumes. For monoculture experiments, biovolume increase was described using a generic primary growth model [42]:


(2) \begin{align*} \frac{1}{N(t)}\frac{dN(t)}{dt}={\mu}_{\mathrm{max}}\bullet \alpha (t)\bullet f(t) \end{align*}

This model defines *μ*_max_ as the exponential growth rate, α(t) as the adjustment function, and *f*(t) as the inhibition function. The nlsMicrobio package was employed to fit the Baranyi & Roberts primary growth model, providing estimates for *μ*_max_, lag phase (derived from α(t)), maximum population size (*N*_max_, derived from f(t)), and initial population size (*N*_0_).

Two types of models were fitted to the competition data between pathogens and bioprotective flora. Model selection between Jameson-type and Lotka-Volterra models for competition conditions was performed based on the Akaike Information Criterion (AIC), with the lower AIC value indicating the model that best balanced goodness of fit and parsimony.

The first type of model is the Jameson-type model [43]. The Jameson effect can be described as competition between species to use environmental resources in order to maximise their growth and population. When the common resource(s) are exhausted, the competition is over and the growth of each species in the population stops.

The growth stops simultaneously by both populations. Both competitors share the inhibition function (*f*(t)) of the exponential growth.


(3) \begin{align*} \frac{1}{N_A(t)}\frac{d{N}_A(t)}{dt}={\mu}_{\max A}\bullet{f}_A(t) \end{align*}

(4) \begin{align*} \frac{1}{N_B(t)}\frac{d{N}_B(t)}{dt}={\mu}_{\max B}\bullet{f}_B(t) \end{align*}

The inhibition function being


(5) \begin{align*} {f}_A(t)={f}_B(t)=\left\{\begin{array}{l}1\ if\ t<{t}_{\mathrm{max}}\\{}0\ if\ t\ge{t}_{\mathrm{max}}\end{array}\right. \end{align*}

where *t*_max_ is the time at which the stationary phase begins for populations A and B.

The second type of model is the Lotka-Volterra model. In this model, the inhibition functions can be described as follow:


(6) \begin{align*} \left\{\begin{array}{l}{f}_A(t)=\left(1-\frac{N_A(t)+{\alpha}_{AB}{N}_B(t)}{N_{\max A}}\right)\\{}{f}_B(t)=\left(1-\frac{N_B(t)+{\alpha}_{BA}{N}_A(t)}{N_{\max B}}\right)\end{array}\right. \end{align*}

where the parameters *α_AB_* and *α_BA_* are the coefficients of interaction measuring the effects of one species on the other.

It makes no specific assumptions about the dynamics underlying species interactions; they can be parameterised in ways that approximate any combination of underlying mechanisms. For example, in a system with two species, if both *α*_AB_ and *α*_BA_ are <0, species suppress each other's growth, indicating competition.

### Statistical analysis

Results related to the description of biofilm phenotypes were presented as mean and standard deviation (SD). Statistical analysis was performed using two-way ANOVA followed by Fisher's least significant difference without correction, utilising PRISM software (GraphPad, USA, California). Statistical significance was determined at a *P <* .05. The significance levels are denoted as follows: ^*^ for *P <* .05, ^**^ for *P* < .01, ^***^ for *P* < .001, ^****^ for *P* < .0001.

## Results

### Selection of antagonistic strains with high pathogen exclusion potential

**Figure 2:**
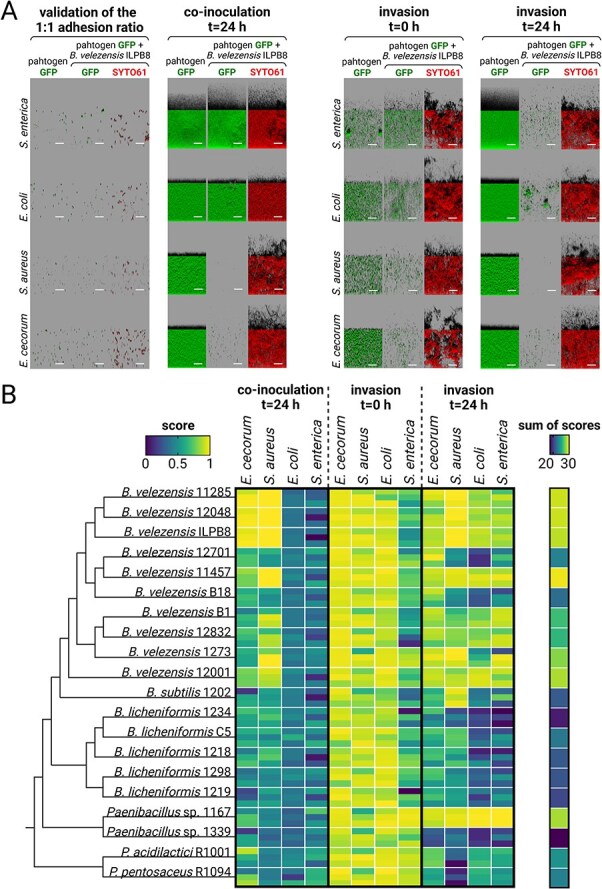
Scores of the antagonistic strain candidates against pathogens in two co-incubation models. (A) Representative images are shown from the strain *B. velezensis* ILPB8. The 2D projections of biofilms were generated using IMARIS software in blend mode. Scale bar = 40 μm. (B) Pathogens were grouped by model and the 20 antagonistic strain candidates were ordered according to a phylogenetic tree reflecting their relatedness. Pathogen GFP biovolumes were quantified to identify antagonistic strains that reduced pathogen biovolume compared to pathogens growing alone under the same conditions. In the heat map, a score of one represents the highest antagonist activity and 0 represents the lowest, calculated based on pathogen GFP biovolume in the presence of antagonistic strain candidates, normalised to the GFP biovolume of the pathogen growing alone. Each square represents one interaction, displaying the means of three biological replicates, each calculated from four technical replicates. The sum of scores against each pathogen across all models was calculated to determine the total antagonistic power of each antagonistic strain candidate.

A screening process was conducted using two in vitro immersed biofilm models to identify antagonistic strains capable of restricting the growth and establishment of four pathogens, *E. cecorum*, *S. aureus*, *E. coli,* and *S. enterica*. One focuses on competition during simultaneous colonisation (co-inoculation), while the other investigates whether antagonistic biofilm candidates prevent subsequent pathogen adhesion and proliferation (invasion). A collection of 20 antagonistic strain candidate(s) from various isolation origins was first characterised for different surface-biofilm and motility phenotypes highlighting a wide range of phenotypic diversity (Supplementary Fig. S6). To ensure a balanced initial co-inoculation, the adhesion ratio of both antagonistic candidates and pathogenic strains was adjusted and validated using CLSM to target the same amount of both partners (Supplementary Fig. S7). After co-inoculation and 24 h of growth, the raw biovolume of GFP-labelled pathogens from z-stacks were extracted by image analysis (Supplementary Fig. S8). Regarding the invasion assay, the raw biovolumes of the pathogen were quantified to investigate its adhesion (invasion t = 0 h, Supplementary Fig. S9) and growth (invasion t = 24 h, Supplementary Fig. S10) on an established antagonistic biofilm candidate. Representative interactions for each model are shown (Fig. 2A), using *B. velezensis* ILPB8 in confrontation with the different pathogens. Antagonistic scores, ranging from 0 to 1, were calculated from pathogen biovolume in the presence of each antagonistic strain candidate, with one indicating maximal pathogen exclusion by the antagonistic bacterium candidate (Fig. 2B). Our results revealed different modes of interactions between the antagonistic bacterium candidate and the pathogen. For instance, in a co-inoculation assay, *E. coli* and *S. enterica* were not excluded. Conversely, in the invasion assay, the *B. velezensis* strains as well as *Paenibacillus* spp. 1167 exhibited a strong capacity to exclude *S. enterica*. Pathogen adhesion to pre-established antagonistic biofilm candidate (i.e. invasion at t = 0 h) was reduced with most strains, except for *S. enterica*. Overall, *B. velezensis* consistently demonstrated superior pathogen exclusion capacity across the two interaction models in comparison to other tested strains. Remarkably, *B. velezensis* strains 11 457, 11 285, 12 048, and ILPB8 consistently achieved the highest scores and illustrated superior performance against *E. cecorum* and *S. aureus*.

Cell counts were performed using *B. velezensis* ILPB8 strain, which exhibits a high antagonistic score, thereby confirming that the reduction in the GFP signal in our experiments was a direct result of a decline in cell numbers (Supplementary Fig. S11).

The macrocolony model was used as an alternative biofilm system to assess the antagonistic potential of the strains against undesirable biofilm formation (Supplementary Fig. S12). Exclusion trends are consistent with those obtained with CLSM in the t = 24 h invasion model.

### Designing antagonistic SynComs via compatibility-guided biofilm assembly

**Figure 3:**
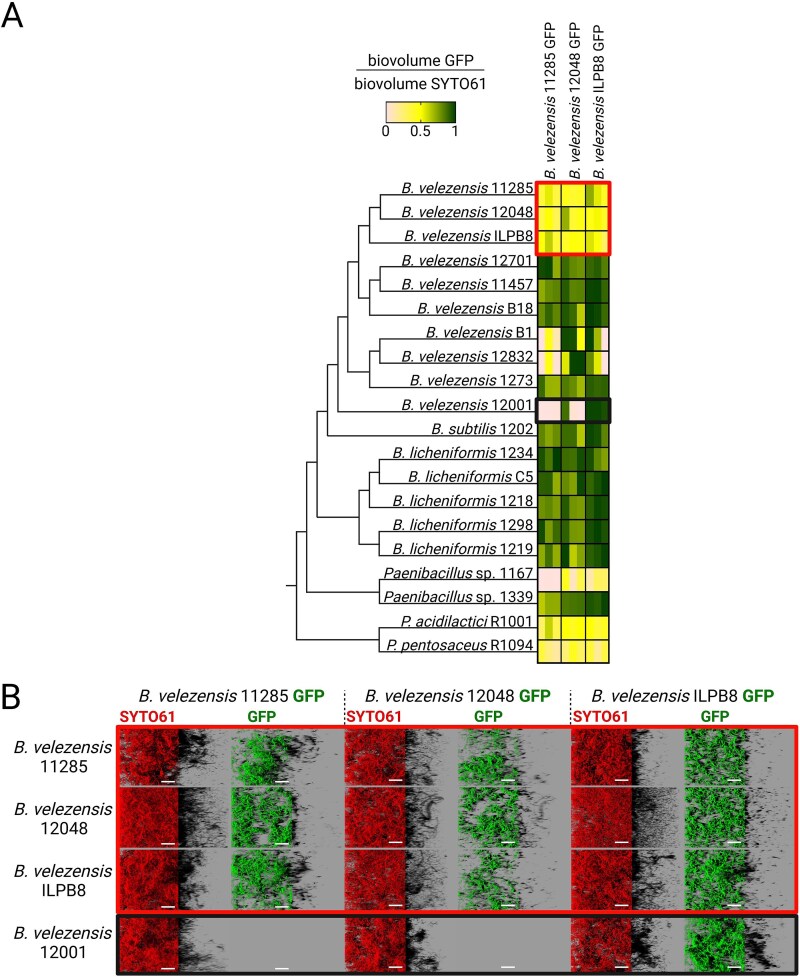
Competitive interaction between GFP-labelled *B. velezensis* and other antagonistic strain candidates. (A) The ability of three *B. velezensis* strains, expressing GFP and exhibiting high antagonistic activity against pathogens, to form mixed submerged biofilms with other strain candidates was evaluated using the co-inoculation model. Strains were ordered according to their phylogenetic distance. The SYTO61 and GFP signals were quantified to calculate the GFP biovolume to SYTO61 biovolume. A GFP/SYTO61 ratio of 0.5, represented in yellow on the figure, indicates a biofilm where the GFP signal (from the green-labelled strain) is equal to the signal exclusively stained with SYTO61 (red-labelled strain). In this scenario, one strain is labelled with GFP and SYTO61 (appearing both green and red), while the other strain is labelled only with SYTO61 (appearing red). A ratio of 0.5 therefore represents equal contributions from both strains, implying coexistence in the biofilm. Each square represents one interaction and shows the means of three biological replicates, each derived from four technical replicates. Red and black rectangles highlight the examples illustrated below. (B) Representative examples illustrate the interactions between the three *B. velezensis* GFP strains capable of forming two-strain mixed biofilms. The exclusion scenario is demonstrated by *B. velezensis* 12 001, which excludes *B. velezensis* 11 285 and *B. velezensis* 12 048, while it is excluded by *B. velezensis* ILPB8. The 2D projections of biofilms were generated using IMARIS software in blend mode. Scale bar = 40 μm.

*B. velezensis* strains 11 285, 12 048, and ILPB8 were selected based on their high cumulative antagonistic scores against pathogens and their competence for GFP labelling. Although *B. velezensis* 11 457 achieved the highest score, it was not used in further experiments due to its inability to be transformed. We employed a co-inoculation model to identify compatible mixed biofilms resulting from interactions between the three GFP-tagged *B. velezensis* strains and other antagonistic candidate strains. Initially, the adhesion ratios between *B. velezensis* 12 048 GFP and 20 bacterial isolates were determined and applied subsequently to *B. velezensis* 11 285 GFP and *B. velezensis* ILPB8 GFP (Supplementary Fig. S13). After 24 h of growth, the biovolumes were extracted from the z-stacks of images. The GFP/SYTO61 ratio was calculated to assess the strains’ ability to coexist or exclude each other (see Fig. 3A and B). Our results suggest that selected bacterial isolates exhibit mutual exclusion tendencies. Mixed biofilm formation occurred only when the three GFP-labelled *B. velezensis* strains were combined with their genetically unlabelled counterparts or with the two phylogenetically more distant *Pediococcus* spp. Notably, none of the combinations exhibited a total biovolume (SYTO61) significantly different from that of either strain when grown individually (Supplementary Fig. S14).

**Figure 4:**
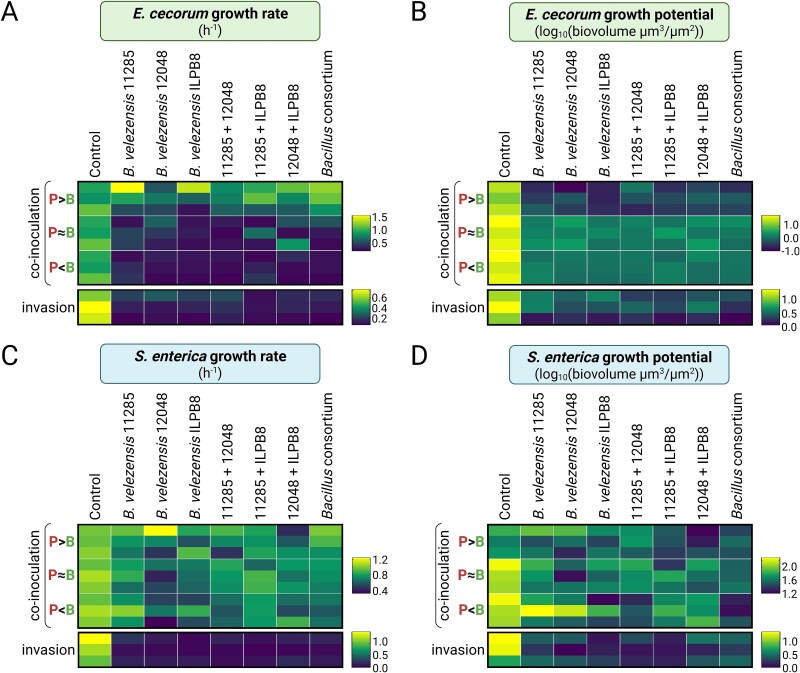
Mixed biofilm composed of three compatible strains of *B. velezensis*. (A) Representative images of mixed biofilms using *B. velezensis* ILPB8 strains wild type, or genetically labelled with GFP or mCherry, along with the addition of DAPI in the biofilm to visualise the entire population in blue. The 2D projections of biofilms were generated using IMARIS software with the easy 3D mode. Scale bar = 30 μm. (B) The percentage of signal from GFP-, mCherry- and DAPI-labelled cells relative to the total biovolume of DAPI in biofilms of the three strains is presented. The sum of the GFP and mCherry biovolumes, subtracted from the total DAPI biovolume, allows for the estimation of the proportion of the nongenetically labelled strain within the mixed biofilm. (C) The co-inoculation model was employed using each wild-type strains, either alone or in combination, and the biofilms were labelled with SYTO9 and observed using the Leica SP8 HCS-CLSM to investigate their overall structure. Biovolume data were extracted and are shown in the graph. (D) Substratum coverage quantification of wild-type strains alone or in consortia is presented. Error bars correspond to standard deviation.

Further studies were carried out to assess the potential of the selected *B. velezensis* strains 11 285, 12 048 and ILPB8, to form a three-strain mixed biofilm. Co-inoculation experiments were performed, incorporating two of the *B. velezensis* strains, genetically tagged with GFP or mCherry, together with the WT of the third *B. velezensis* strain (Fig. 4A). After staining the consortium with the DNA binding fluorescent dye DAPI, the observations and proportion quantifications of the three strains revealed a uniformly mixed biofilm (Fig. 4B). The strains within the *Bacillus* consortium showed no significant differences in their biovolume (Fig. 4C). However, the *Bacillus* consortium was found to exhibit a significantly higher surface area coverage than the one- and two-strains combinations (Fig. 4D).

### Pre-established antagonistic biofilms enhance pathogen exclusion

**Figure 5:**
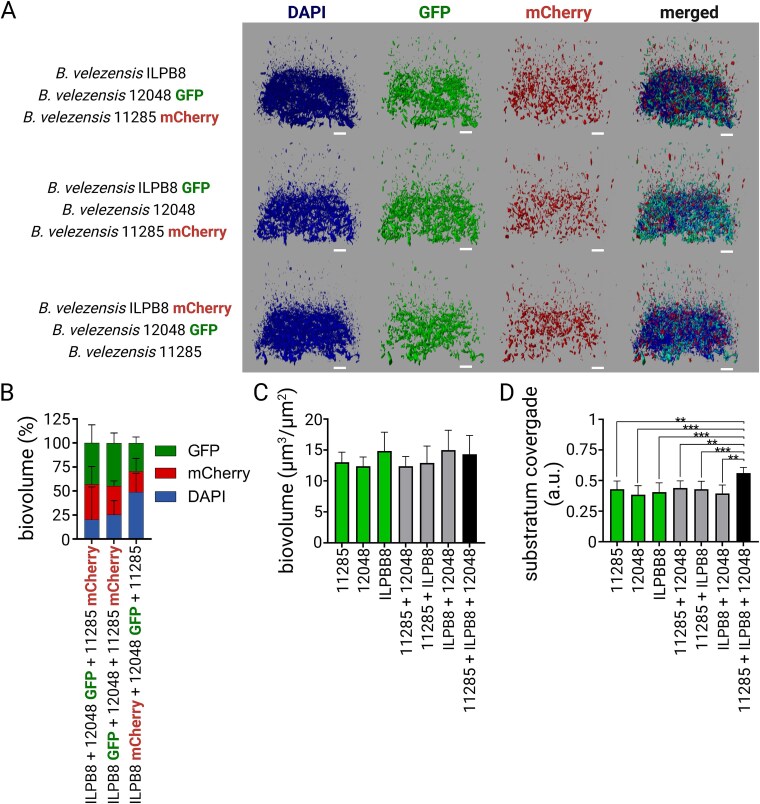
4D CLSM experiment to measure pathogen growth parameters in mixed biofilms with *B. velezensis*. (A) Growth rate (h^−1^) determination of *E. cecorum* by modelling GFP biovolume curves and (B) calculation of growth potential for *E. cecorum* determined by subtracting the initial biovolume N_0_ from the final biovolume N_f_ (calculated in log10 (biovolume μm^3^/μm^2^)). The initial biovolume ratios of *E. cecorum* GFP to *B. velezensis* were determined at the start of the experiment (P for ‘pathogen’ and B for ‘*Bacillus*’: ratio P > B = 1.4 (+/− 0.2), ratio P ≈ B = 0.3 (+/− 0.06), ratio P < B = 0.03 (+/− 0.02), invasion = 0.2 (+/− 0.04)). (C) Growth rate determination of *S. enterica* by modelling GFP biovolume curves and (D) calculation of growth potential for *S. enterica* determined by subtracting the initial biovolume N_0_ from the final biovolume N_f_ (calculated in log10 (biovolume μm^3^/μm^2^)). The initial biovolume ratios of *S. enterica* GFP to *B. velezensis* were determined at the start of the experiment (ratio P > B = 3.2 (+/− 0.8), ratio P ≈ B = 0.4 (+/− 0.1), ratio P < B = 0.1 (+/− 0.05), invasion = 4.8 (+/− 0.8)). The *Bacillus* consortium corresponds to the SynCom of *B. velezensis* strains 11 285, 12 048 and ILPB8. Each square represents one interaction performed using the HCS mode of the CLSM and shows the means of three biological replicates, each calculated from four technical replicates.

Exploration of the dynamics underlying the exclusion of *E. cecorum* and *S. enterica* by *B. velezensis* was conducted using HCS-CLSM kinetic experiments applied to the two co-incubation models (Supplementary Fig. S15). To achieve this, the vital membrane-dye FM4–64 was used to visualise the entire biofilm without altering the growth of the pathogen (Supplementary Fig. S16). Biovolume curves over time were extracted from the images and processes using the Jameson or Lotka-Volterra mathematical models to determine the growth rate and the growth potential (Supplementary Fig. S17). Here, growth potential refers to the change in biovolume over time, calculated as the difference between final and initial biovolume (N_f_ - N_0_), reflecting the strains net expansion within the biofilm under specific conditions. To reflect ecologically relevant scenarios in which antagonistic strains and pathogens encounter each other at varying proportions, and to improve our understanding of their interaction dynamics, we applied different initial adhesion ratios between *B. velezensis* and the pathogens. The first ratio (ratio P > B) targeted twice the number of pathogens in comparison to the antagonistic strain(s), the second ratio (ratio P ≈ B) twice more antagonistic strain(s) compared to the pathogens and the third ratio (ratio P < B) 10 times the amount of the antagonistic bacteria candidate(s). We observed that the growth rate of *E. cecorum* was reduced in the presence of *B. velezensis* under all conditions except when the initial ratio started with a higher proportion of the pathogen (ratio P > B) (Fig. 5A). For all ratios, the growth potential was found to decrease and even to become negative with the ratio P > B, indicating a decline of *E. cecorum* biovolume at the end of the kinetics compared to the initial situation (Fig. 5B). In this specific case, the interactions were found to adhere to a Lotka-Volterra model. The growth rate and growth potential of *S. enterica* in the presence of *B. velezensis* were only observed to be altered in the context of invasion (Fig. 5C and D). Except for the ratio P > B in co-inoculation with *E. cecorum*, the interaction profiles were found to adhere to a Jameson model for all the strains of *B. velezensis* and their associated consortia against pathogens. However, significant differences in exclusion capacity between the *B. velezensis* strains against the two pathogens could be observed, highlighting a specificity between strains (Supplementary Fig. S18, S19). An important observation is that *Bacillus* consortia composed of multiple strains do not exhibit greater pathogen exclusion than individual strains, and their effect is not superior to that of the most effective single strain.

### Additive exclusion of *Salmonella enterica* by pre-established mixed-species biofilms of *Bacillus velezensis* and *Pediococcus* spp.

**Figure 6:**
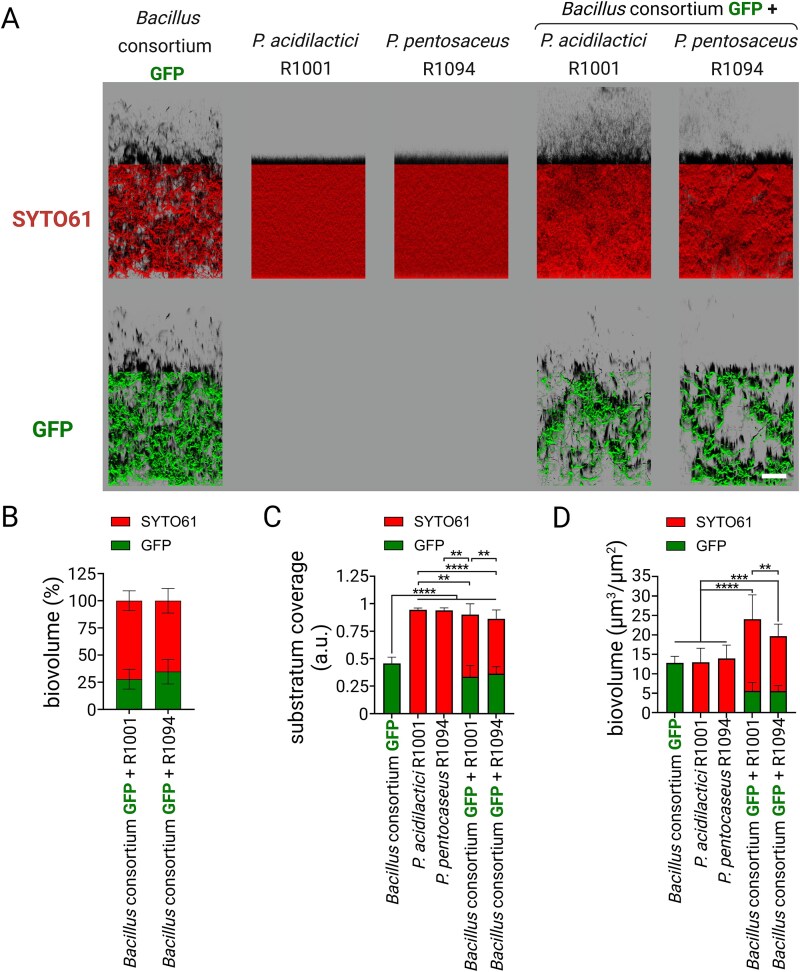
Compatible mixed biofilm composed of the *Bacillus* consortium with *Pediococcus* spp. (A) Representative images showing three compatible strains of *B. velezensis* GFP (*Bacillus* consortium: strains 11 285 GFP, 12048 GFP, and ILPB8 GFP) co-cultured together with *Pediococcus* spp. SYTO61 was used to label all the population in red, while *B. velezensis* were marked with GFP. The 2D projections of biofilms were generated using IMARIS software in blend mode. Scale bar = 50 μm. (B) Percentage of signal from GFP and SYTO61, indicating the proportion of each strain within the biofilm. (C) The co-inoculation model was used to investigate the overall structure of the biofilm. Biovolume data were extracted and are presented in the graph. (D) Substratum coverage quantification of strains alone or in consortia, illustrating the extent of surface coverage by each configuration. Error bars represent standard deviation.

In order to expand the diversity of the consortia, we studied mixed biofilms formed by the *Bacillus* consortium and two strains of *Pediococcus* spp. Each *Pediococcus* strain was selected for its compatibility with the specific *Bacillus* strain within the consortium. Initially, a co-inoculation model was used to investigate the formation of mixed biofilms formed between the *Bacillus* consortium and each of the two *Pediococcus* spp*.* strains. After 24 h of co-growth, confocal imaging showed that the strains formed a mixed biofilm with no evidence of competitive exclusion (Fig. 6A and B). The four-strain consortia covered over 80% of the surface, mainly due to the influence of *Pediococcus* spp. biofilm, which developed between the clusters formed by the *Bacillus* consortium (Figs 6A–C), and exhibited a greater biovolume compared to controls (Fig. 6D).

**Figure 7:**
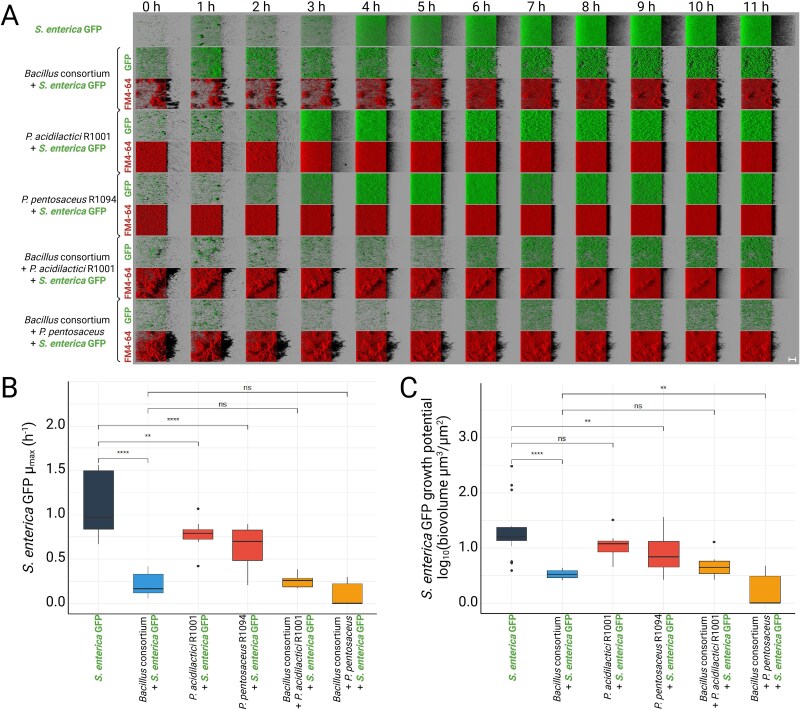
Characterisation of antagonistic activity against pathogens by consortia of *B. velezensis* and *Pediococcus* spp. in the invasion model. (A) Representative CLSM images showing biofilm growth over time for each condition. The green colour corresponds to GFP labelling of *S. enterica*, while the red colour represents total population labelling by FM4–64. The SynCom of *B. velezensis* strains 11 285, 12 048 and ILPB8 (*Bacillus* consortium) was co-cultured with *P. acidilactici* R1001 or with *P. pentosaceus* R1094 before the adhesion of *S. enterica* GFP in the invasion model. The initial biovolume ratios of *S. enterica* GFP to the antagonistic strains were determined at the start of the experiment (*Bacillus* consortium = 6.3 (+/− 2.2), R1001 = 0.14 (+/− 0.05), R1094 = 0.44 (+/− 0.20), *Bacillus* consortium + R1001 = 0.20 (+/− 0.03), *Bacillus* consortium + R1094 = 0.40 (+/− 0.09)). The 2D projections of biofilms were generated using IMARIS software in blend mode. Scale bar = 40 μm. (B) Box plot modelling of *μ*_max_ of *S. enterica* GFP in the invasion model, showing statistical analysis of the additive effects of *B. velezensis* and *Pediococcus* spp. consortia (x + y) on pathogen growth inhibition. Error bars represent standard deviation. (C) Box plot modelling of growth potential of *S. enterica* GFP in the invasion model, showing statistical analysis of the additive effects of *B. velezensis* and *Pediococcus* spp. consortia (x + y) on pathogen growth inhibition. Error bars represent standard deviation.

The most effective exclusion activity against pathogens at 24 h is achieved by mixed biofilms composed of *Pediococcus* spp. and *B. velezensis* strains from the *Bacillus* consortium (Supplementary Fig. S20A). Indeed, the exclusion effect of two-species biofilms (one *B. velezensis* + one *Pediococcus* spp.) was compared to each single strain alone. The consortia composed of *B. velezensis* and *Pediococcus* spp. demonstrated a consistently enhanced ability to exclude *S. enterica* at 24 h compared to that of individual antagonistic strains in the invasion model (*P* < .0001). In addition, the consortia performed at least as well as the best individual strains (Supplementary Figs S20B–G). Therefore, we investigated the dynamics of *S. enterica* exclusion in the first 12 h of invasion using HCS-CLSM coupled with modelling (Fig. 7A). The growth rate of *S. enterica* was found consistently reduced in the presence of antagonistic strains, with no improvement observed in the *Bacillus* consortium when combined with *Pediococcus* spp. (Fig. 7B). However, a significant (*P* < 0.01) decrease in *S. enterica* growth potential was observed when *P. pentosaceus* R1094 was added to the *Bacillus* consortium, compared to the performance of either the *Bacillus* consortium or *P. pentosaceus* R1094 alone (Fig. 7C). *S. enterica* was enumerated under the same conditions (Supplementary Fig. S21). In the presence of *Pediococcus* spp. alone, *S. enterica* levels were comparable to the control, suggesting a densification of the biofilm rather than exclusion of the pathogen. In contrast, when the *Bacillus* consortium was present, either alone or in combination with *Pediococcus* spp., a reduction of more than 6 log₁₀ colony-forming unit (CFU)/ ml was observed.

We investigated whether the acidification of the environment by *Pediococcus* spp. was involved in the observed enhancing effect of *B. velezensis* on the exclusion of *S. enterica* in the invasion t = 24 h model (Supplementary Fig. S22). We demonstrated that acidification proportionally reduced the biovolume of *S. enterica* biofilms but did not affect the exclusion by *B. velezensis* ILPB8 (Supplementary Fig. S23).

Interestingly, the biofilm lifestyle influences interactions differently compared to the planktonic lifestyle. Indeed, in the planktonic mode, *Pediococcus* spp. are excluded by *B. velezensis* (Supplementary Fig. S24). *S. enterica* systematically excludes *B. velezensis* or *Pediococcus* spp. when starting with the same ratio of the two partners in planktonic cultures. Furthermore, using the same initial inoculum as in the invasion model but cultivating under planktonic conditions with agitation, *B. velezensis* cultures completely inhibited *S. enterica* growth, whereas *Pediococcus* spp. alone was excluded (Supplementary Fig. S25).

## Discussion

Common methods for studying microbial interactions in multispecies communities, such as metabarcoding [44], optical density measurements [45], or CFU counting [46], often disrupt intricate 3D structures crucial in biofilm studies [47]. In contrast, our approach utilised nondestructive imaging techniques, allowing precise quantification of individual community members and observation of their spatial arrangement over time. This approach addresses significant gaps in existing methodologies, which often neglect critical factors such as adhesion rates and the formation of filamentous structures or aggregates [1, 25, 48].

Distinguishing the contributions of each strain within biofilms, without disrupting its structure, composed of more than three species presents significant challenges. To overcome these hurdles, genetic manipulation of strains for the expression of distinct fluorophores or the use of specific dyes is necessary. These technical limitations restricted our ability to explore all potential strain combinations, such as co-culturing *P. acidilactici* and *P. pentosaceus,* due to the absence of corresponding fluorescent strains. Population dynamics could be monitored using species-specific qPCR assays to address this objective. Furthermore, this study focuses on interactions between strains within a submerged biofilm model, which only forms under specific physicochemical conditions found in nature. While this model allows for high-throughput 3D analysis, it may not fully reflect biofilm behaviour on surfaces or at air-liquid interfaces. To diversify situations, the macro-colony model was used to study interactions between two colonies developing in a biofilm model at the air-surface interface [49]. Interaction results closely align with those from the T = 24 h invasion model, likely because both involve interactions between pre-organised biofilm communities. Throughout the study, we demonstrate that both the ratio between interacting partners and the timing of pathogen introduction relative to the antagonistic biofilm are critical factors that must be considered when analysing microbial interactions [50]. Future investigations should consider the microbiota context of the studied ecosystem, as it may influence observed interaction phenomena [51, 52].

Through temporal imagining and mathematical modelling, we correlate the type of dynamics driving pathogen exclusion within biofilms, primarily involving spatial and nutritional competition, along with the potential synthesis of antimicrobial compounds. Our findings suggest that pathogen exclusion is largely governed by the Jameson effect, coupled with a specific interference dynamic, where both the growth potential (nutritional competition) and the growth rate (secretion of bacteriostatic molecules) of the pathogen are reduced [16]. While this image-based approach provides a general framework for understanding these dynamics, it does not pinpoint specific molecular effectors responsible for pathogen exclusion. Future studies using omics approaches in combination with genetic analysis may help to elucidate these molecular underpinnings of pathogen exclusion [49].

The inhibitory effect of *B. velezensis* on a broad range of bacteria is well documented, often attributed to the secretion of interfering molecules [53], some of which are specifically expressed in biofilms [54]. Interestingly, *B. velezensis* excludes *E. cecorum* through interference dynamics when the pathogen is initially dominant, a behaviour consistent with Lotka-Volterra dynamics where mutual is observed [22]. However, this effect disappears when *B. velezensis* is present in equal or greater amounts. These findings underscore the critical influence of initial population ratios on the outcome and dynamics of microbial interactions. The biofilm of *B. velezensis* would secrete a molecule capable of killing *E. cecorum,* although the specific nature of this molecule remains unknown. These findings emphasise the need to consider the biofilm lifestyle, as their related kinetics differ significantly from those in planktonic cultures, even when starting with the same initial ratios of antagonistic strain candidates and the evaluated pathogen. This distinction is crucial, as metabolic processes vary widely between biofilm and planktonic states [5]. Therefore, the biofilm lifestyle should be a key consideration in designing SynComs intended for environments where bacteria are likely to form biofilms.

Before evaluating the antagonistic effects of SynComs on pathogen growth, we systematically assessed the compatibility of strains to establish stable mixed biofilms without excluding other partners. Kovačec E *et al.* showed that candidate probiotic strains of *B. subtilis*, selected for their ability to exclude *Salmonella enterica* Typhimurium, can mutually inhibit each other, leading to a reduction in the overall inhibitory effect against the pathogen [55]. Similarly, our results revealed that *B. velezensis* strains, selected for their ability to exclude pathogens, also tend to exclude other *Bacillus* spp. However, the compatibility between the three *B. velezensis* strains is consistent with the principle of kin compatibility, whereby organisms differentiate between genetically related (kin) and unrelated (nonkin) individuals [56, 57]. This discrimination is likely based on the detection of small signalling molecules or specific flagellin motifs in *B. velezensis* [58]. To validate the hypothesis of compatibility among *Bacillus* strains through kin discrimination, genome comparisons as well as compatibility tests of swarming profiles could be performed.

Notably, only the three selected competitive *B. velezensis* strains were able to form mixed biofilms with one another and with the more distantly related *Pediococcus* spp. Even so, no significant increase in pathogen exclusion was observed in these compatible consortia compared to individual strains, likely due to the presence of shared competition mechanisms. By selecting more phylogenetically distant but compatible species, such as *Pediococcus* spp., we have achieved genetic diversification, enhanced exclusion mechanisms and extended coverage to different physico-chemical environments [52]. Interaction experiments confirmed that consortia composed of *B. velezensis* and *Pediococcus* spp*.* exhibited antagonistic scores against pathogens at least as high as the best-performing individual strain. Additionally, consortia of *B. velezensis* and *Pediococcus* spp. demonstrated a systematic additive effect against *S. enterica* in the invasion model, where the antagonistic biofilm is pre-established before pathogen arrival. This effect is not only attributed to pH reduction by *Pediococcus* spp., but rather to spatial and nutritional competition. In addition, mixed biofilms formed by *B. velezensis* and *Pediococcus* spp. exhibited greater surface coverage and biovolume than single-species biofilms. This increased biomass likely enhances their competitive fitness and contributes to greater exclusion of *S. enterica* through both spatial occupation and resource depletion. Kinetic experiments over 12 h in the invasion model demonstrate that the *Bacillus* consortium, when paired with the *P. pentosaceus* R1094 strain, significantly reduces the growth potential of *S. enterica*. This suggests that the combination of the *Bacillus* consortium with the R1094 strain allows for more effective early exclusion, which levels out over time with the *P. acidilactici* R1001. Further studies should investigate potential metabolic exchanges between *B. velezensis* and *Pediococcus* spp. through the use of omics approaches [59] and genome-based modelling [60].

In conclusion, our study highlights the compatibility between selected *B. velezensis* and *Pediococcus* spp. strains, resulting in increased biofilm formation and improved pathogen elimination with a specific additive effect on *S. enterica*. The compatibility and exclusion capacity of strains are intricately linked to biofilm formation, emphasising the importance of pre-establishing antagonistic biofilms on surfaces to achieve additive effects. The imaging methodology provides a framework for assembling SynComs with strain compatibility and antagonistic potential against pathogens. Moreover, the techniques developed in our study have broad potential applications in biotechnological fields aimed at pathogen control, bioremediation, biopreservation, and probiotics.

## Supplementary Material

Supplementary_information_GUENEAU_et_al_2025_ycaf156

## Data Availability

Raw stacks of images dataset corresponding to Fig. 2, 3 has been deposited in Data INRAE (accession number https://doi.org/10.57745/XRXQEI). Further inquiries can be directed to the corresponding author.

## Code availability

R codes used to assess growth parameters are available on Github: https://github.com/lguillier/dataset_kin
