# Correction to: 3D imaging-driven assembly of multispecies biofilms with antagonistic activity against undesirable bacteria

**DOI:** 10.1093/ismeco/ycaf222

**Published:** 2025-12-15

This is a correction to: Virgile Guéneau, Laurent Guillier, Cécile Berdous, Marie-Françoise Noirot-Gros, Guillermo Jiménez, Julia Plateau-Gonthier, Pascale Serror, Mathieu Castex, Romain Briandet, 3D imaging-driven assembly of multispecies biofilms with antagonistic activity against undesirable bacteria, *ISME Communications*, Volume 5, Issue 1, January 2025, https://doi.org/10.1093/ismeco/ycaf156

In the originally published version of the manuscript the images for Figures 4 and 5 were erroneously transposed. These are now corrected in the article so that the correct images for Figures 4 and 5 are matched to the correct captions.

