# Supplementary material for: 3D imaging-driven assembly of multispecies biofilms with antagonistic activity against undesirable bacteria
Source: ISME Commun. 2025 Sep 5;5(1):ycaf156. doi: 10.1093/ismeco/ycaf156 (PMC12539569; doi:10.1093/ismeco/ycaf156)
Supplement: Supplementary_information_GUENEAU_et_al_2025_ycaf156 [file supplementary_information_gueneau_et_al_2025_ycaf156.pdf]

Supplementary informations

Supplementary Figure 1: Characteristics of strains used in this study.

Name	Origine and genotype	Reference
Bacillus velezensis 11285	unknown	Lallemand Aquapharm
Bacillus velezensis 12048	unknown	Lallemand Aquapharm
Bacillus velezensis ILPB8	Surfaces of commercial broiler chicken houses	[1]
Bacillus velezensis 12701	unknown	Lallemand Aquapharm
Bacillus velezensis 11457	unknown	Lallemand Aquapharm
Bacillus velezensis B18	Surfaces of commercial broiler chicken houses	[1]
Bacillus velezensis B1	Surfaces of commercial broiler chicken houses	[1]
Bacillus velezensis 12832	unknown	Lallemand Aquapharm
Bacillus velezensis 1273	Very wet soil of a snow combe, 2400m altitude	INRAE B3D
Bacillus velezensis 12001	unknown	Lallemand Aquapharm
Bacillus subtilis 1202	Water from a river	INRAE B3D
Bacillus licheniformis 1234	Small greenish pebbles	INRAE B3D
Bacillus licheniformis C5	Surfaces of commercial broiler chicken houses	[1]
Bacillus licheniformis 1218	Sea urchin intestine in sea water	INRAE B3D
Bacillus licheniformis 1298	Pinkish grey gravel	INRAE B3D
Bacillus licheniformis 1219	Greyish sandy soil	INRAE B3D
Paenibacillus sp. 1167	Black soil	INRAE B3D
Peanibacillus sp. 1399	Dark brown soil	INRAE B3D
Pediococcus acidilactici R1001	unknown	Lallemand
Pediococcus pentosaceus R1094	unknown	Lallemand
Bacillus velezensis 11285 mCherry	*Bacillus velezensis* 11285 with pGM11-*mcherry* (ery)	This study
Bacillus velezensis 12048 GFP	*Bacillus velezensis* 12048 with pCM11-*gfp* (ery)	This study
Bacillus velezensis ILPB8 GFP	*Bacillus velezensis* ILPB8 with pCM11-*gfp* (ery)	This study
Bacillus velezensis ILPB8 mCherry	*Bacillus velezensis* ILPB8 with pGM11-*mcherry* (ery)	This study
Staphylococcus aureus RN4220	Laboratory strain with pALC2084-*gfp* (ery)	[2]
Enterococcus cecorum DSM20682	Strain from DSM collection, isolated from chicken caecum with pCM11-*gfp* (ery)	This study
Salmonella enterica enterica serotype *enteritidis* NCTC6676	Strain from NCTC collection, isolated from a dead cow with pCM11-*gfp* (amp)	This study
Escherichia coli CIRMBP0248	Strain from CIRMBP collection, isolated from chicken intestine with pCM11-*gfp* (amp)	This study

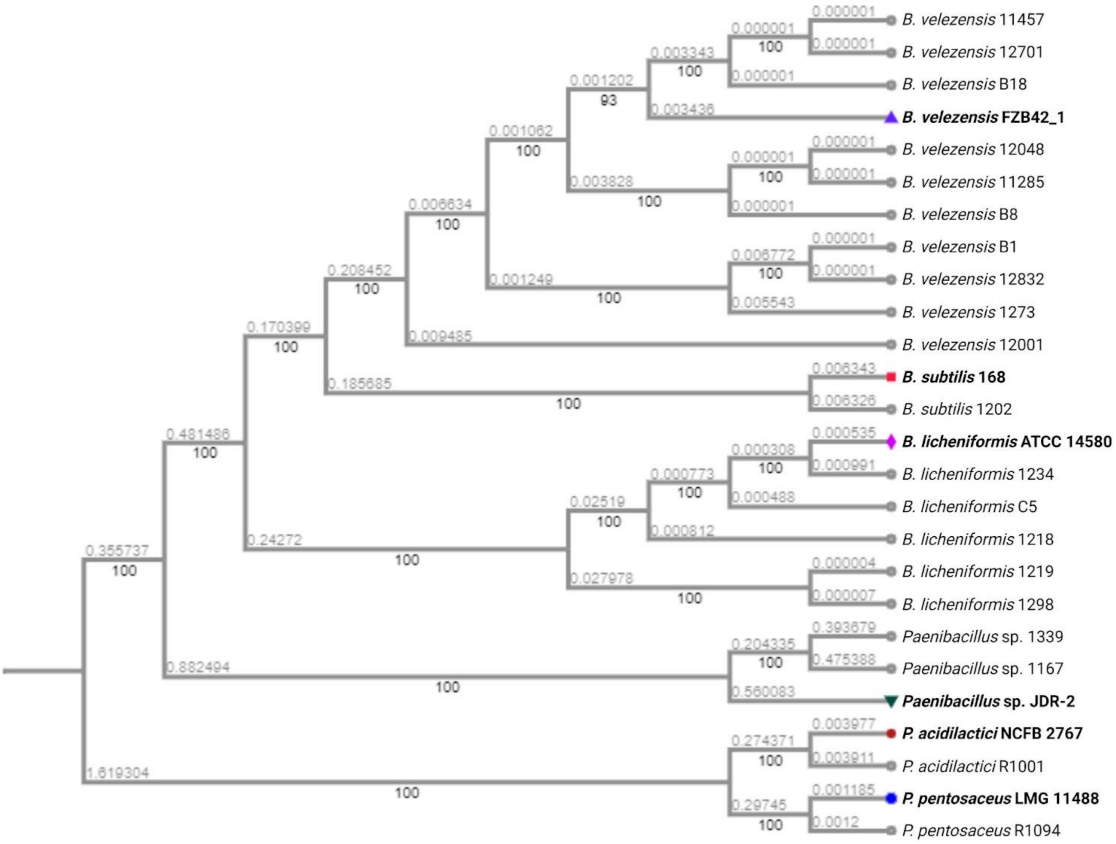

Supplementary Figure 2: Phylogenetic tree of the 20 candidate antagonistic strains used in the study, along with reference strains. The tree was obtained using the Bacterial Phylogenetic Tree Service of BV-BRC [3]**.** Reference strains have been added to the tree in bold.

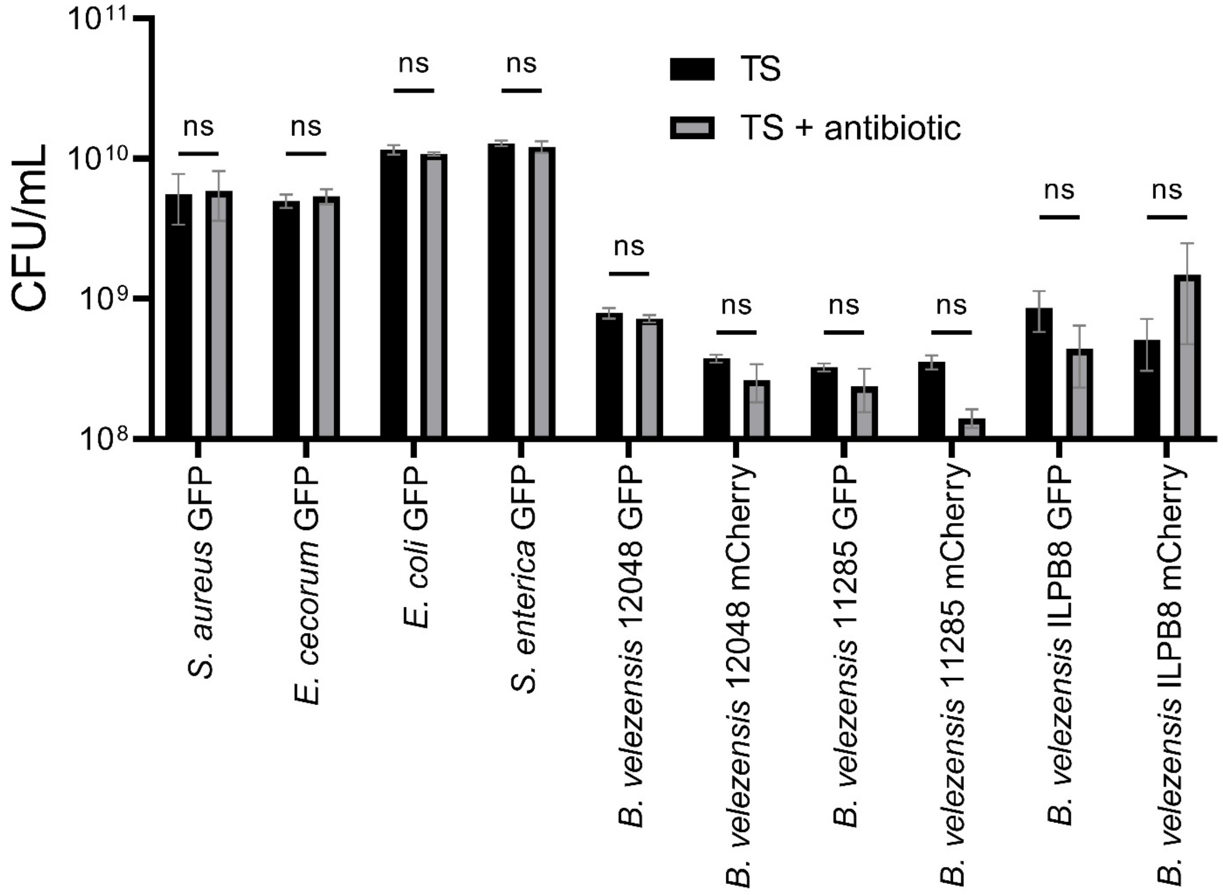

Supplementary Figure 3: Plasmid stability in biofilm experiments. Biofilms were cultured in 96-well plates in 200 µL of TSB for 24 hours without antibiotics from overnight cultures of bacteria initially grown in the presence of antibiotics. Post-cultivation, biofilms were detached and homogenised in their related wells. Plasmid stability was assessed by plating on TSA or TSA supplemented with antibiotics (ery 5 µg/mL for *S. aureus* and *E. cecorum* and amp 100 µg/mL for *E. coli* and *S. enterica*). Three biological replicates were performed for each condition. Bacterial counts were compared using the Wilcoxon matched-pairs signed-rank test (GraphPad Prism). Error bars correspond to the standard deviation of the measurements.

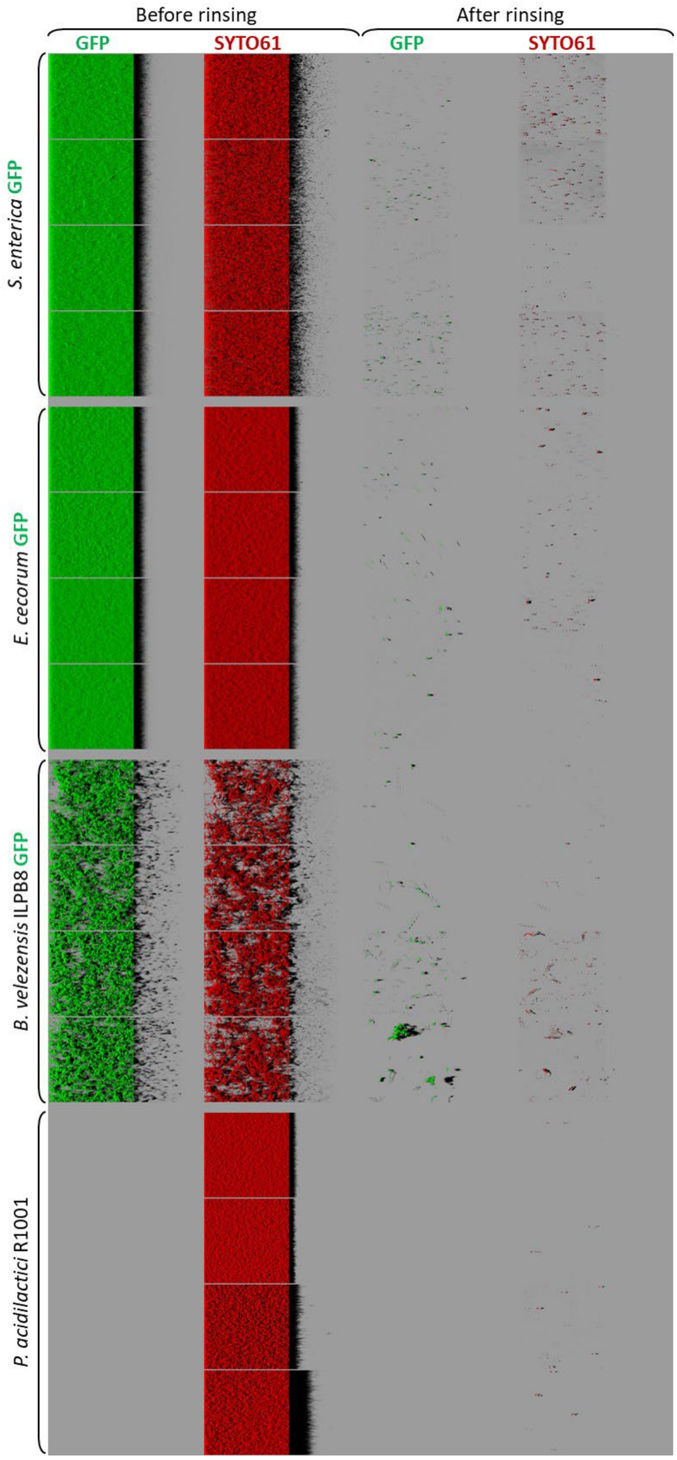

Supplementary Figure 4: Validation procedure following mechanical detachment of biofilms. Representative biofilms from the study were grown for 24 hours at the bottom of 96-well plates in TSB. Biofilms were stained with SYTO61. CLSM (confocal laser scanning microscopy) imaging was performed before and after the mechanical detachment procedure to assess biofilm removal. Four wells per condition were analysed.

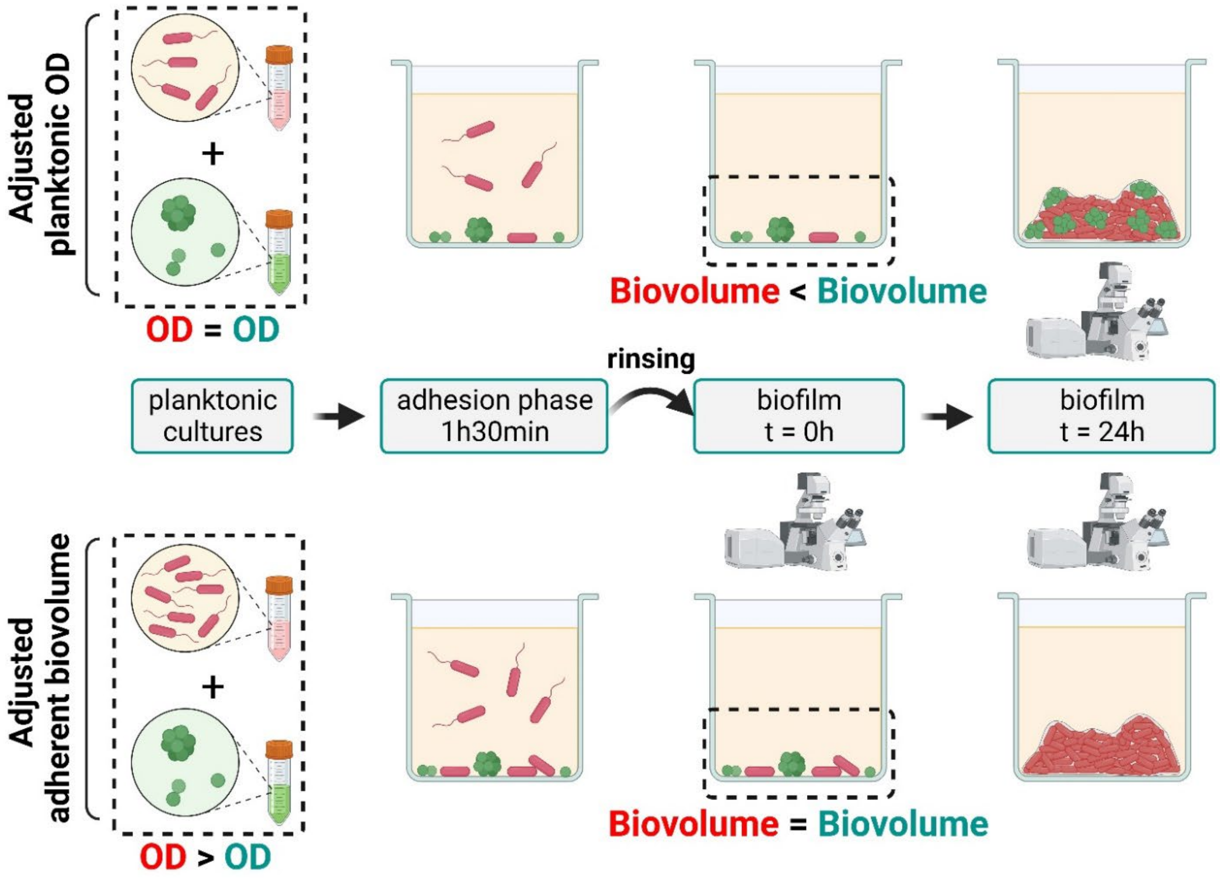

Supplementary Figure 5: Method used to standardise the initial inoculum for co-incubation experiments. The method used adjusts the volumes of planktonic overnight cultures to achieve an identical biovolume for both partners at the start of the experiment after the adhesion step. This method accounts for potential aggregate formation and differences in adhesion rate.

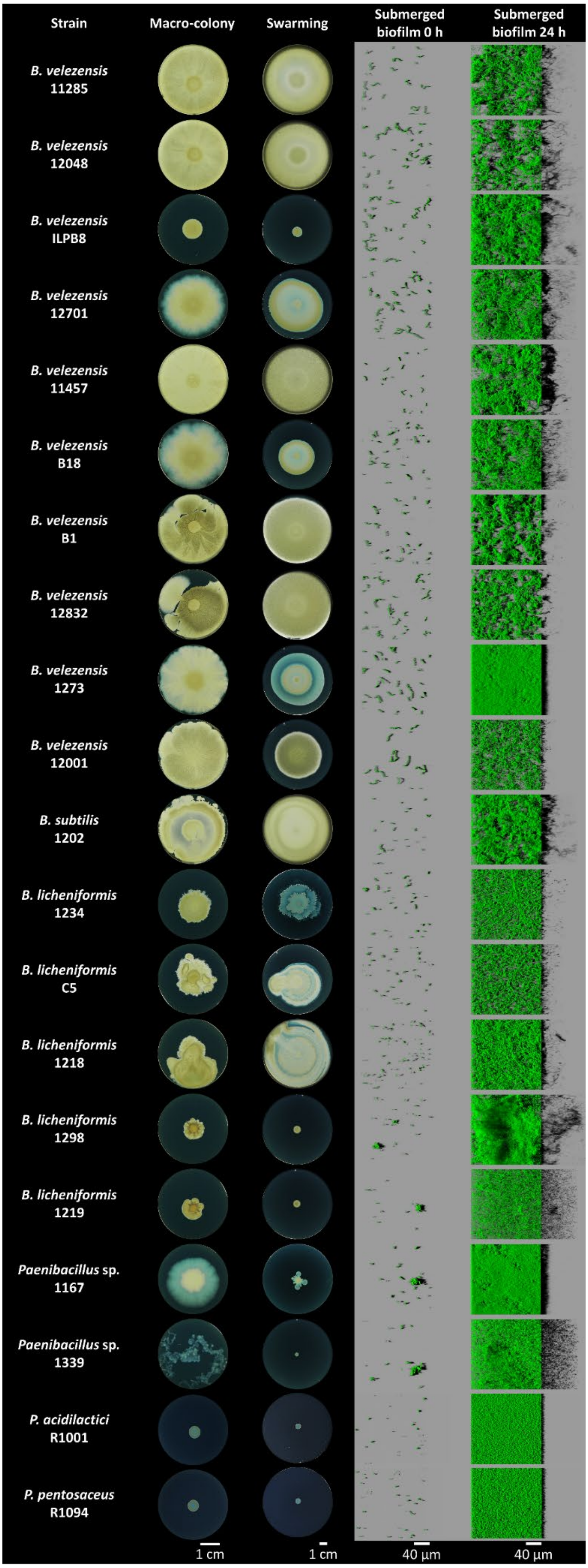
Strain
Macro-colony
Swarming
Submerged biofilm 0 h
Submerged biofilm 24 h
B. velezensis 11285
B. velezensis 12048
B. velezensis ILPB8
B. velezensis 12701
B. velezensis 11457
B. velezensis B18
B. velezensis B1
B. velezensis 12832
B. velezensis 1273
B. velezensis 12001
B. subtilis 1202
B. licheniformis 1234
B. licheniformis C5
B. licheniformis 1218
B. licheniformis 1298
B. licheniformis 1219
Paenibacillus sp. 1167
Paenibacillus sp. 1339
P. acidilactici R1001
P. pentosaceus R1094
1 cm
1 cm
40 µm
40 µm

Supplementary Figure 6: Phenotypic characterisation of *Bacillus* strains in different biofilm models. To initiate the macro-colony model experiments, 5 ml cultures were prepared in trypticase soy broth (TSB; BioMérieux, France) from glycerol stock stored at -80°C. These cultures were incubated at 30°C overnight without agitation. Following homogenisation by rapid vortexing for 5 seconds, 3 µL of culture were transferred to a well of a six-well plate containing 4 ml of TSA 1.5% agar. The samples were dried under a hood for 10 minutes and incubated at 30°C for 4 days. For the swarming experiment, a similar protocol was followed, but with 20 mL of TSA 0.7% agar in a Petri dish, incubated overnight at 30°C. Mono-species submerged biofilms were observed using the CLSM. Overnight cultures were diluted at 1:100e in TSB, and 200 µL of this dilution was added to each well of a 96-well plate. The cultures were allowed to adhere for 1 hour and 30 minutes. After the adhesion phase, the supernatant was carefully removed and replaced with fresh TSB. The plates were then incubated for 24 hours at 30°C without agitation, and 432 image stacks were collected. Representative images are shown. The two-dimensional projections of biofilms were generated using IMARIS software in blend mode.

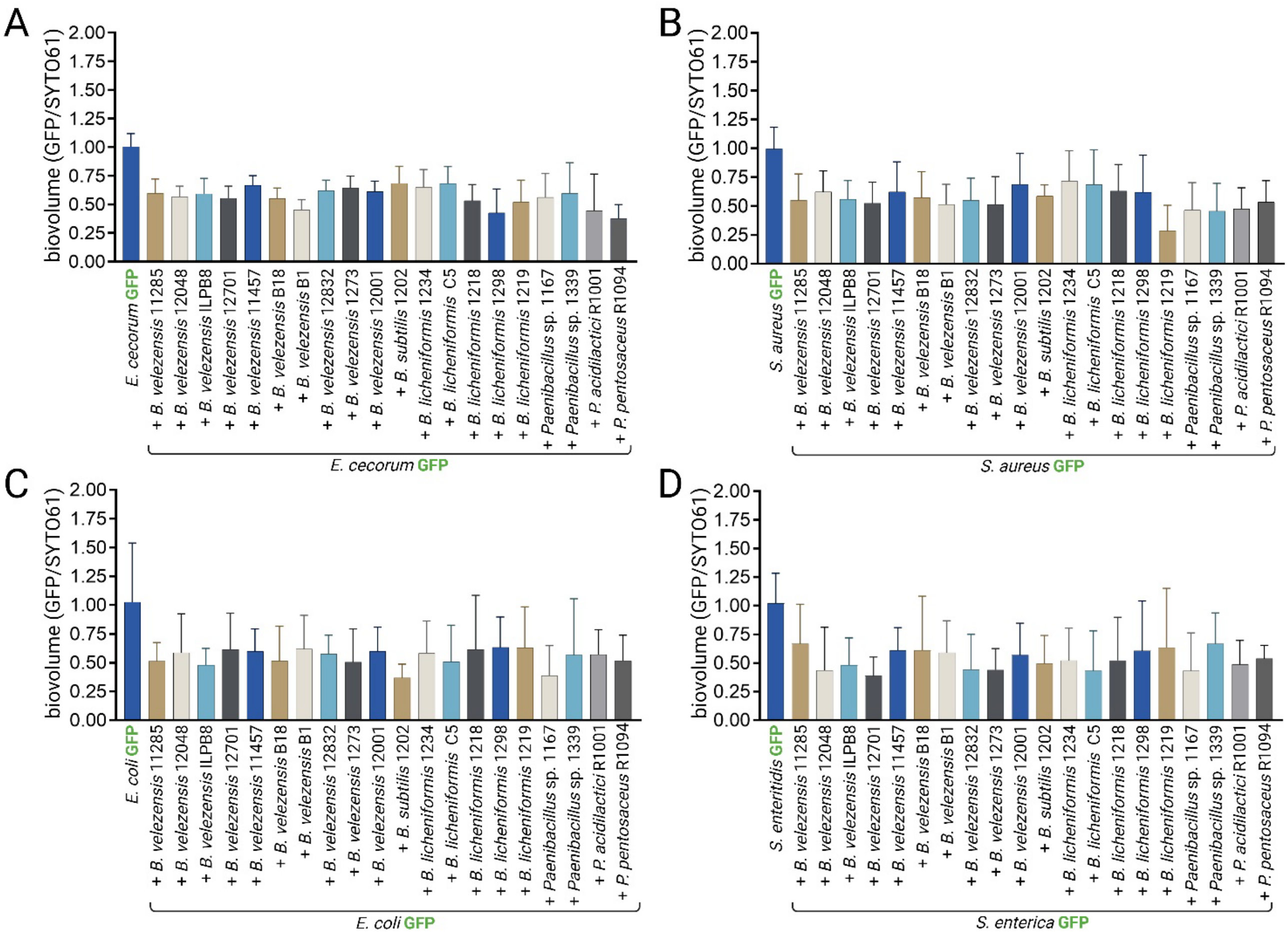

Supplementary Figure 7: Adhesion ratio of candidate antagonistic strains and GFP-labelled pathogens in the co-inoculation growth model. Volumes of overnight cultures of non-labelled antagonistic strain candidates were adjusted to achieve an equal biovolume of GFP-labelled pathogens and antagonistic strains adhered to the bottom of the wells. Pathogens were genetically marked with GFP, and the entire population was chemically labelled with SYTO61 after 1 h 30 min of adhesion. A GFP biovolume to SYTO61 biovolume ratio of 0.5 indicates an equal quantity of *B. velezensis* 12048 GFP and antagonistic strain.

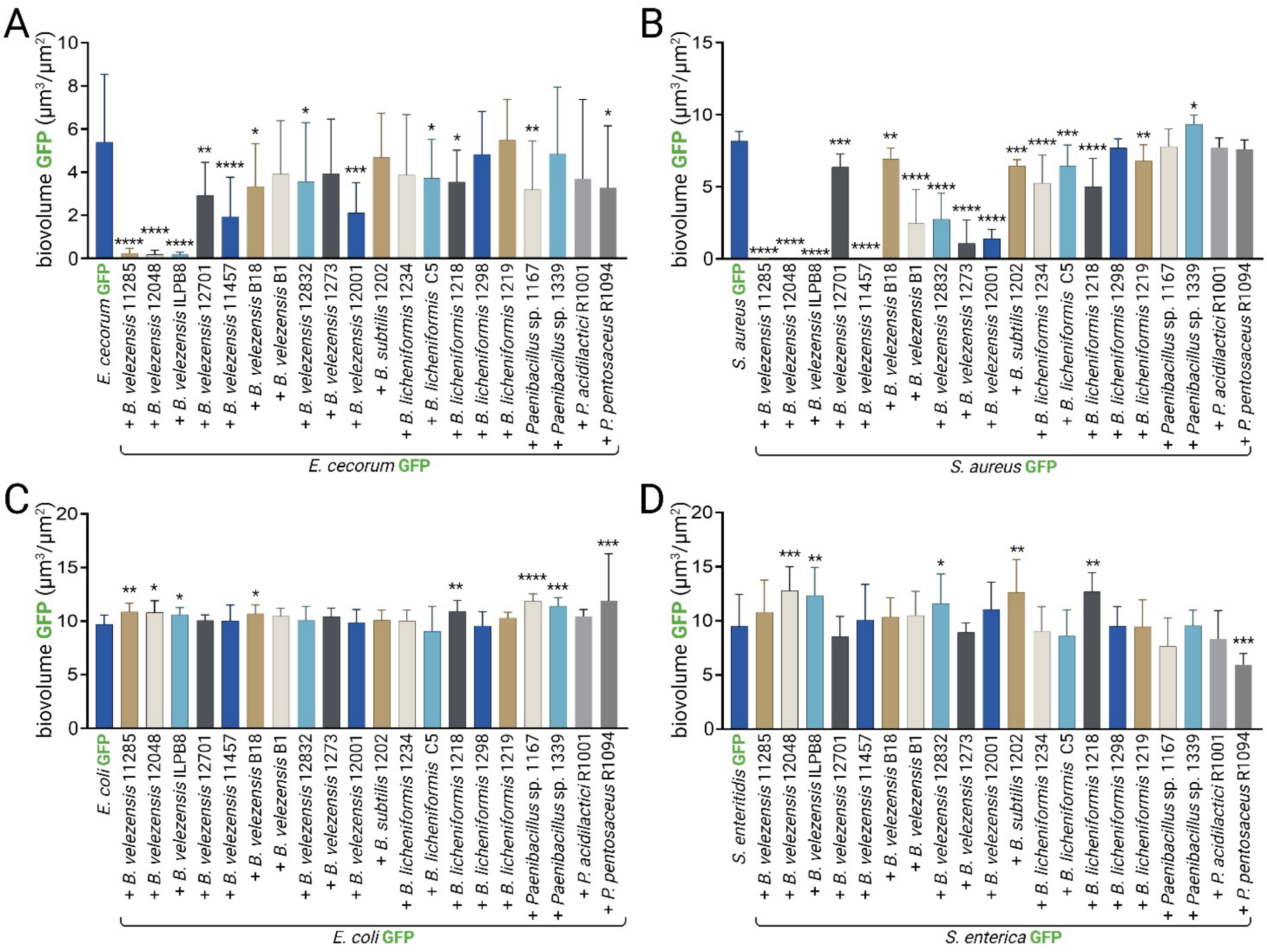

Supplementary Figure 8: Biovolume of GFP-labelled pathogens in the co-inoculation growth model with candidate antagonistic strains. The biovolume of GFP-labelled pathogens co-cultured with candidate antagonistic strains was measured. The results are shown for (A) GFP-labelled *E. cecorum*, (B) GFP-labelled *S. aureus*, (C) GFP-labelled *E. coli*, and (D) GFP-labelled *S. enterica*. Error bars correspond to standard deviation. The biovolume of the GFP-labelled pathogen in the presence of the candidate antagonistic strain was compared to the biovolume of the pathogen grown alone.

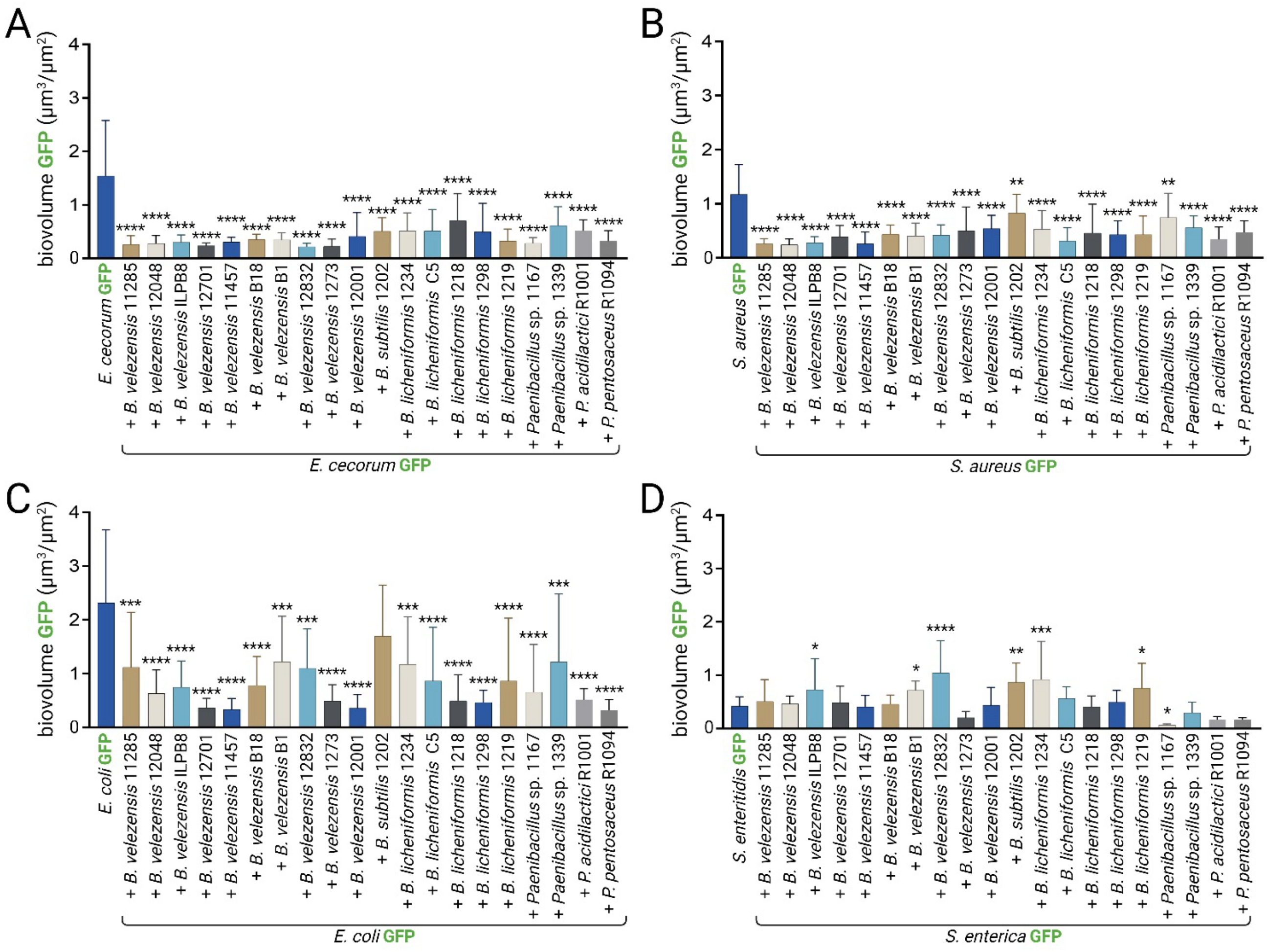

Supplementary Figure 9: Biovolume of GFP-labelled pathogen in the invasion t=0 h growth model co-cultured with candidate antagonistic strains. The biovolume of GFP-labelled pathogens in the invasion t=0 h growth model, co-cultured with candidate antagonistic strains, was measured. The results are shown for (A) GFP-labelled *E. cecorum*, (B) GFP-labelled *S. aureus*, (C) GFP-labelled *E. coli*, and (D) GFP-labelled *S. enterica*. Error bars correspond to standard deviation. The biovolume of the GFP-labelled pathogen in the presence of the candidate antagonistic strains was compared to the biovolume of the pathogen grown alone.

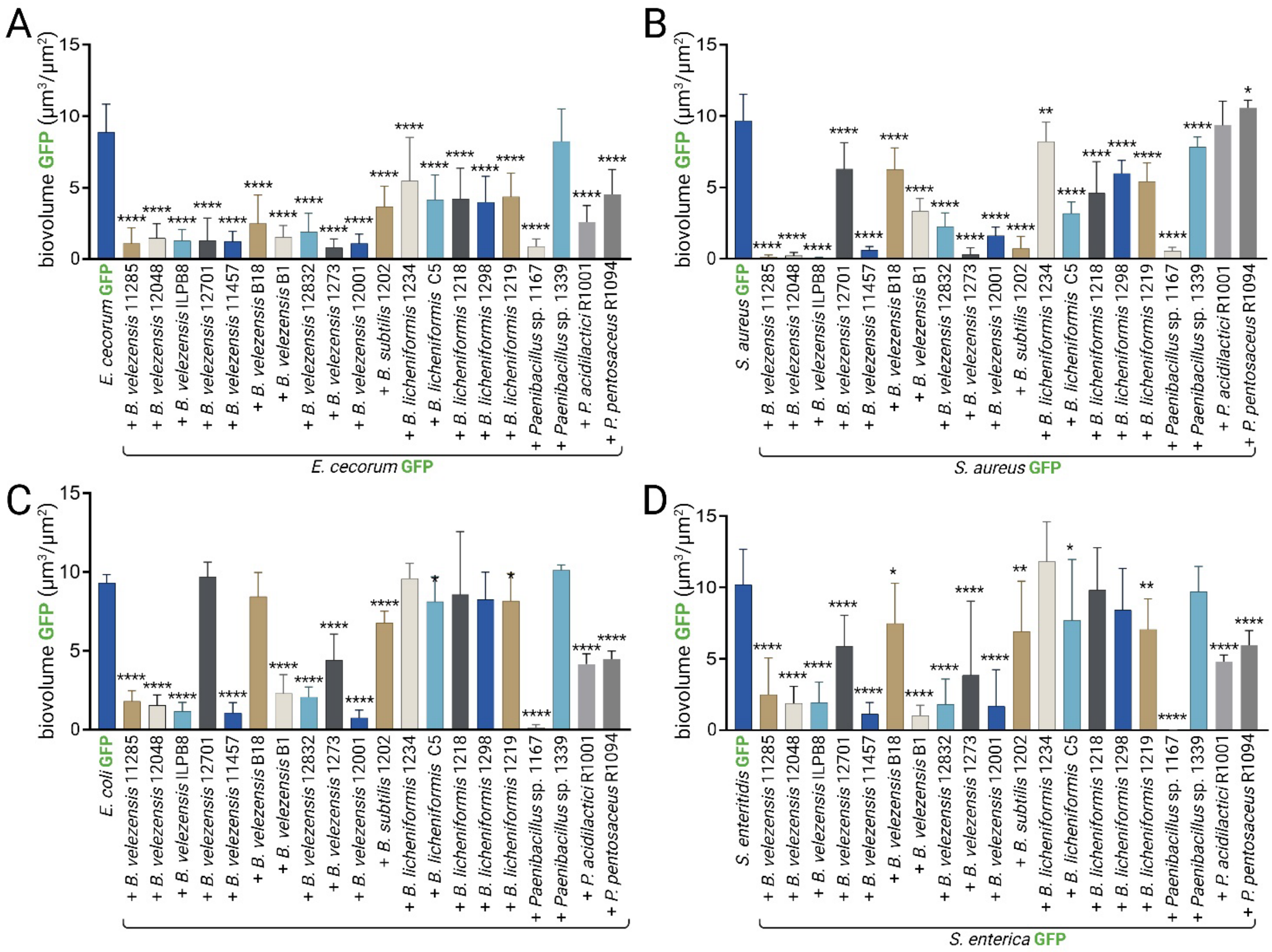

Supplementary Figure 10: Biovolume of GFP-labelled pathogen in the invasion t=24 hours growth model co-cultured with candidate antagonistic strains. The results are shown for (A) GFP-labelled *E. cecorum*, (B) GFP-labelled *S. aureus*, (C) GFP-labelled *E. coli*, and (D) GFP-labelled *S. enterica*. Error bars correspond to standard deviation. The biovolume values of the GFP-labelled pathogen in the presence of the candidate antagonistic strains were compared with the biovolume of the pathogen grown alone.

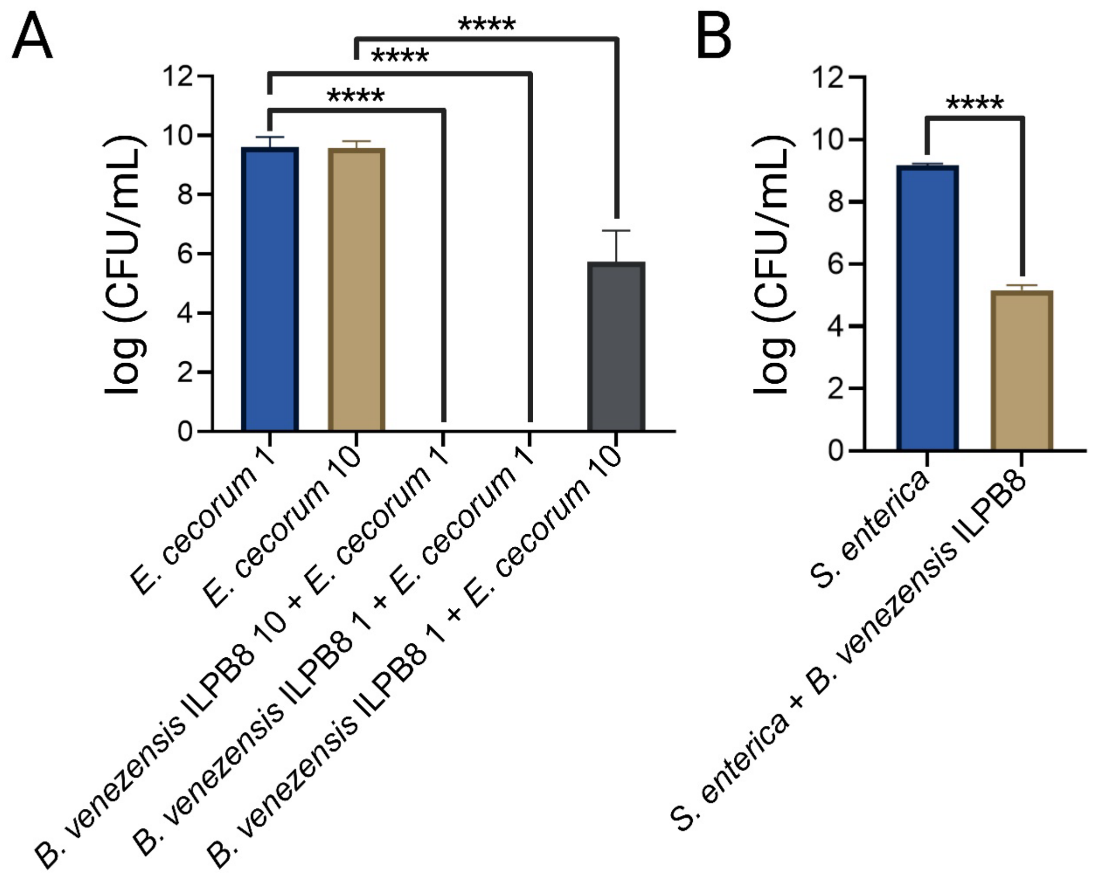

Supplementary Figure 11: Enumeration of pathogens after 24 hours of incubation in the presence or absence of *B. velezensis* ILPB8. Pathogen counts were measured in submerged biofilms after 24 hours of incubation with and without *B. velezensis* ILPB8. Biofilms were vortexed 10 s vigorously before enumeration. (A) Results with *E. cecorum* using different ratios of the two partners at the start of the co-inoculation experiment. (B) Results with *S. enterica* in the invasion model. Experiments were performed with 3 biological replicates. Error bars correspond to standard deviation.

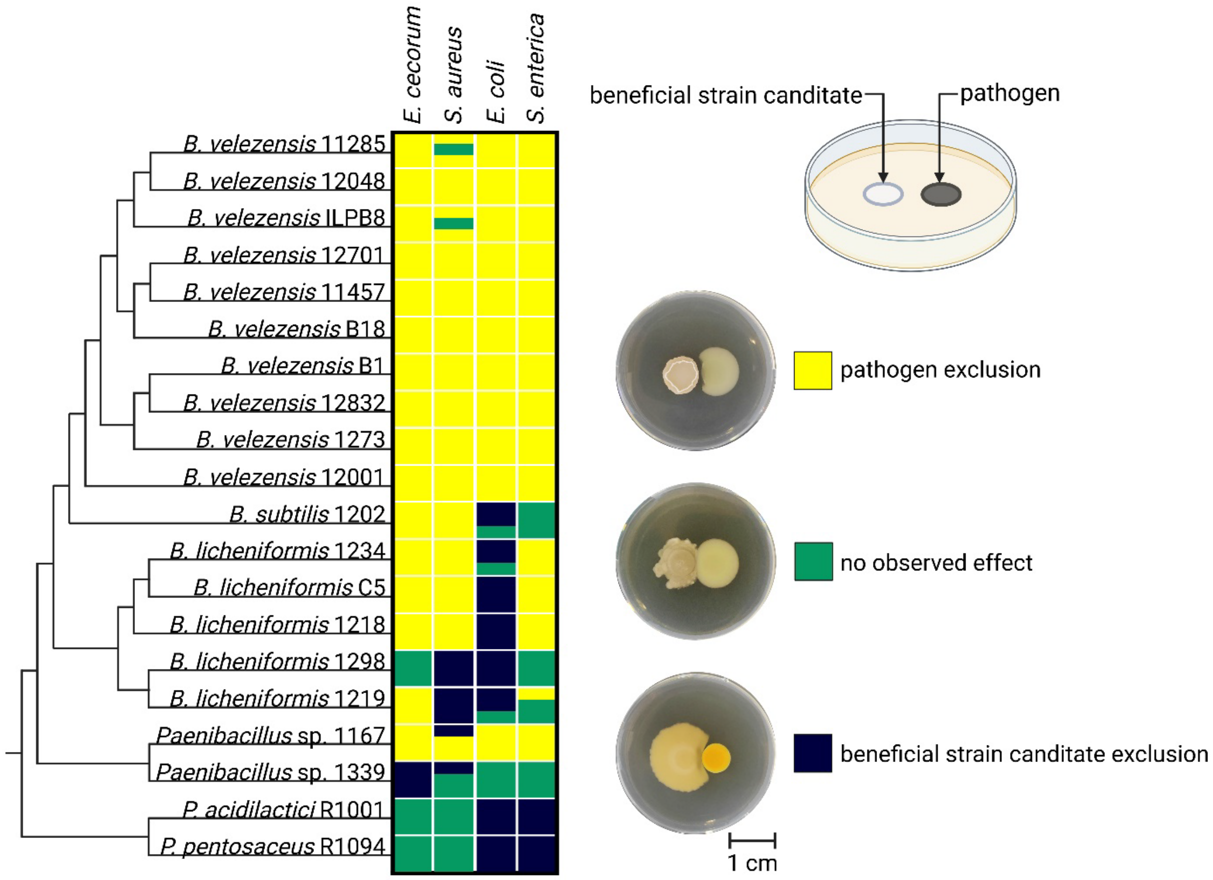

Supplementary Figure 12: Antagonism of the candidate antagonistic strains against pathogens in a macro-colony interaction model. The competition between the two partners in the same well was assessed by observing the colonies. When one partner exhibited an altered colony morphology, it was considered to be excluded by the other partner. When the colonies met without morphological defects in either partner, no conclusion was drawn regarding the exclusion effect of one partner over the other. Three representative examples have been chosen to illustrate the results.

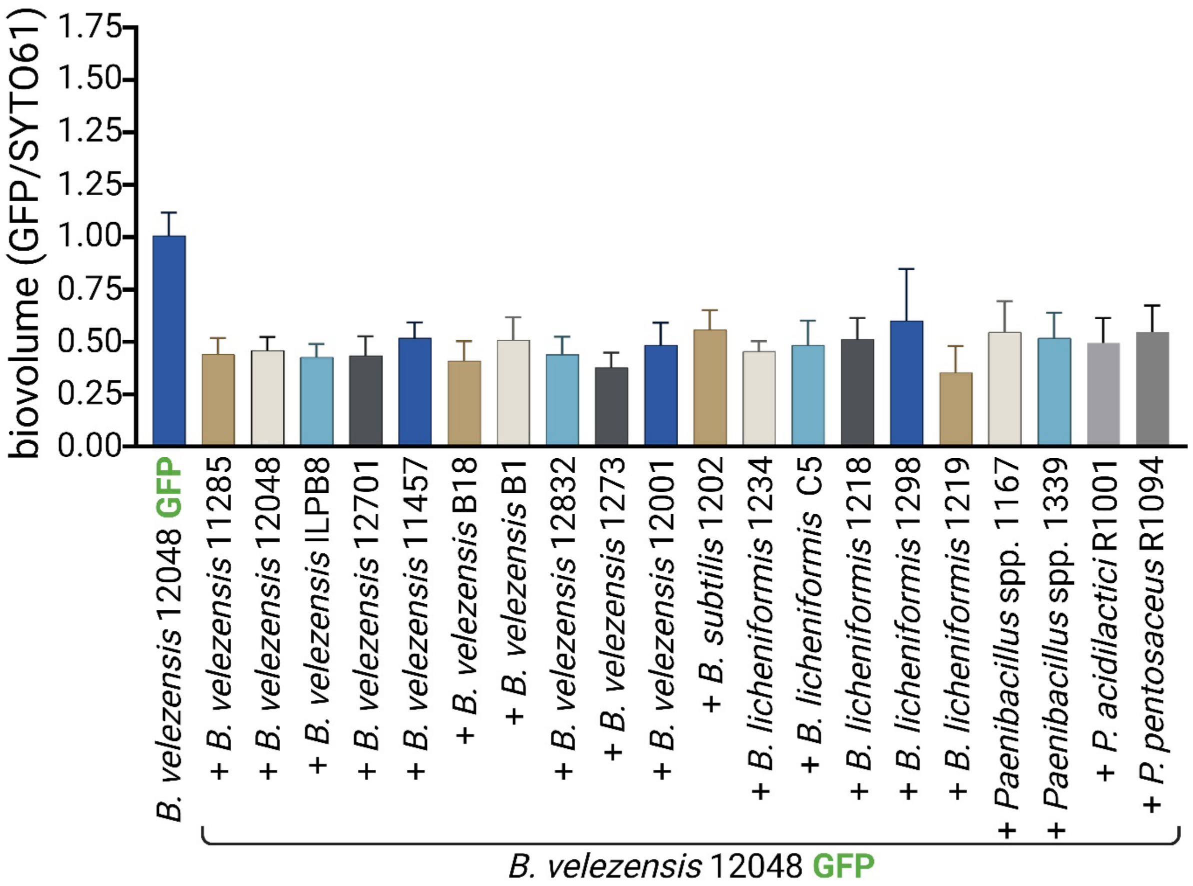

Supplementary Figure 13: Adhesion ratio of the 20 antagonistic strain candidates and GFP-labelled *B. velezensis* in the co-inoculation growth model. (A) Volumes from overnight cultures were calibrated to achieve an equal biovolume of *B. velezensis* GFP and non-labelled *Bacillus* spp. strains adhered to the bottom of the wells. *B. velezensis* 12048 was genetically marked with GFP, and the entire population was chemically labelled with SYTO61 after 1 h 30 min of adhesion. A GFP biovolume to SYTO61 biovolume ratio of 0.5 indicates an equal quantity of *B. velezensis* 12048 GFP and antagonistic strains. (B) Verification of the adhesion ratio was performed with GFP-labelled *B. velezensis* 11285, 12048 and ILPB8 and *Pediococcus* spp. Error bars correspond to standard deviation.

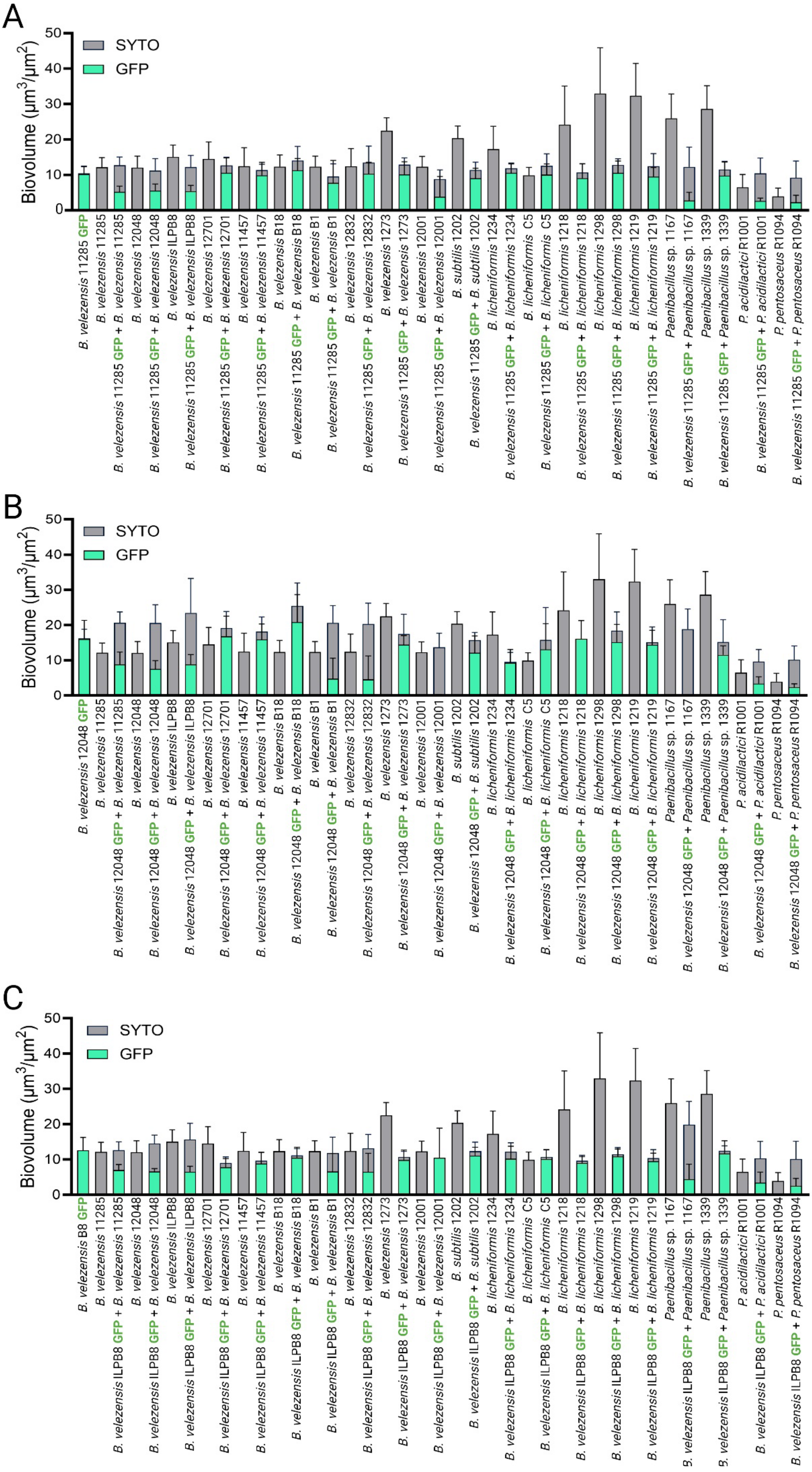
A
B
C
Biovolume (µm³/µm²)
0
10
20
30
40
50
SYTO
GFP
B. velezensis 11285 GFP
B. velezensis 11285
B. velezensis 11285 GFP + B. velezensis 11285
B. velezensis 12048
B. velezensis 11285 GFP + B. velezensis 12048
B. velezensis ILPB8
B. velezensis 11285 GFP + B. velezensis ILPB8
B. velezensis 12701
B. velezensis 11285 GFP + B. velezensis 12701
B. velezensis 11457
B. velezensis 11285 GFP + B. velezensis 11457
B. velezensis B18
B. velezensis 11285 GFP + B. velezensis B18
B. velezensis B1
B. velezensis 11285 GFP + B. velezensis B1
B. velezensis 12832
B. velezensis 11285 GFP + B. velezensis 12832
B. velezensis 1273
B. velezensis 11285 GFP + B. velezensis 1273
B. velezensis 12001
B. velezensis 11285 GFP + B. velezensis 12001
B. subtilis 1202
B. velezensis 11285 GFP + B. subtilis 1202
B. licheniformis 1234
B. velezensis 11285 GFP + B. licheniformis 1234
B. licheniformis C5
B. velezensis 11285 GFP + B. licheniformis C5
B. licheniformis 1218
B. velezensis 11285 GFP + B. licheniformis 1218
B. licheniformis 1298
B. velezensis 11285 GFP + B. licheniformis 1298
B. licheniformis 1219
B. velezensis 11285 GFP + B. licheniformis 1219
Paenibacillus sp. 1167
B. velezensis 11285 GFP + Paenibacillus sp. 1167
Paenibacillus sp. 1339
B. velezensis 11285 GFP + Paenibacillus sp. 1339
P. acidilactici R1001
B. velezensis 11285 GFP + P. acidilactici R1001
P. pentosaceus R1094
B. velezensis 11285 GFP + P. pentosaceus R1094
B. velezensis 12048 GFP
B. velezensis 12048 GFP + B. velezensis 11285
B. velezensis 12048 GFP + B. velezensis 12048
B. velezensis 12048 GFP + B. velezensis ILPB8
B. velezensis 12048 GFP + B. velezensis 12701
B. velezensis 12048 GFP + B. velezensis 11457
B. velezensis 12048 GFP + B. velezensis B18
B. velezensis 12048 GFP + B. velezensis B1
B. velezensis 12048 GFP + B. velezensis 12832
B. velezensis 12048 GFP + B. velezensis 1273
B. velezensis 12048 GFP + B. velezensis 12001
B. velezensis 12048 GFP + B. subtilis 1202
B. velezensis 12048 GFP + B. licheniformis 1234
B. velezensis 12048 GFP + B. licheniformis C5
B. velezensis 12048 GFP + B. licheniformis 1218
B. velezensis 12048 GFP + B. licheniformis 1298
B. velezensis 12048 GFP + B. licheniformis 1219
B. velezensis 12048 GFP + Paenibacillus sp. 1167
B. velezensis 12048 GFP + Paenibacillus sp. 1339
B. velezensis 12048 GFP + P. acidilactici R1001
B. velezensis 12048 GFP + P. pentosaceus R1094
B. velezensis B8 GFP
B. velezensis ILPB8 GFP + B. velezensis 11285
B. velezensis ILPB8 GFP + B. velezensis 12048
B. velezensis ILPB8 GFP + B. velezensis ILPB8
B. velezensis ILPB8 GFP + B. velezensis 12701
B. velezensis ILPB8 GFP + B. velezensis 11457
B. velezensis ILPB8 GFP + B. velezensis B18
B. velezensis ILPB8 GFP + B. velezensis B1
B. velezensis ILPB8 GFP + B. velezensis 12832
B. velezensis ILPB8 GFP + B. velezensis 1273
B. velezensis ILPB8 GFP + B. velezensis 12001
B. velezensis ILPB8 GFP + B. subtilis 1202
B. velezensis ILPB8 GFP + B. licheniformis 1234
B. velezensis ILPB8 GFP + B. licheniformis C5
B. velezensis ILPB8 GFP + B. licheniformis 1218
B. velezensis ILPB8 GFP + B. licheniformis 1298
B. velezensis ILPB8 GFP + B. licheniformis 1219
B. velezensis ILPB8 GFP + Paenibacillus sp. 1167
B. velezensis ILPB8 GFP + Paenibacillus sp. 1339
B. velezensis ILPB8 GFP + P. acidilactici R1001
B. velezensis ILPB8 GFP + P. pentosaceus R1094

Supplementary Figure 14: GFP-labelled *B. velezensis* biovolume in the co-inoculation growth model co-cultured with 20 antagonistic candidate strains. The biovolume of GFP-labelled *B. velezensis* co-cultured with 20 antagonistic candidate strains was measured. The results are shown for (A) GFP-labelled *B. velezensis* 11285, (B) GFP-labelled *B. velezensis* 12048, and (C) GFP-labelled *B. velezensis* ILPB8. Error bars correspond to standard deviation.

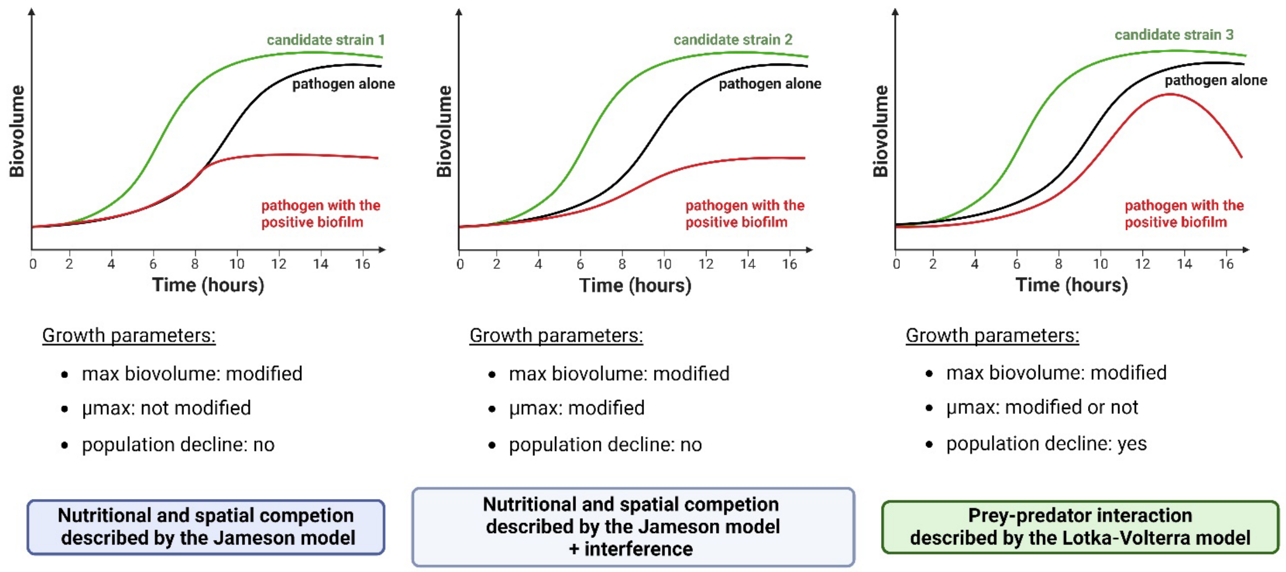

Supplementary Figure 15: Dynamics of biofilm formation through CLSM kinetic studies. Schematic representation of potential interactions at CLSM and the resulting dynamics.

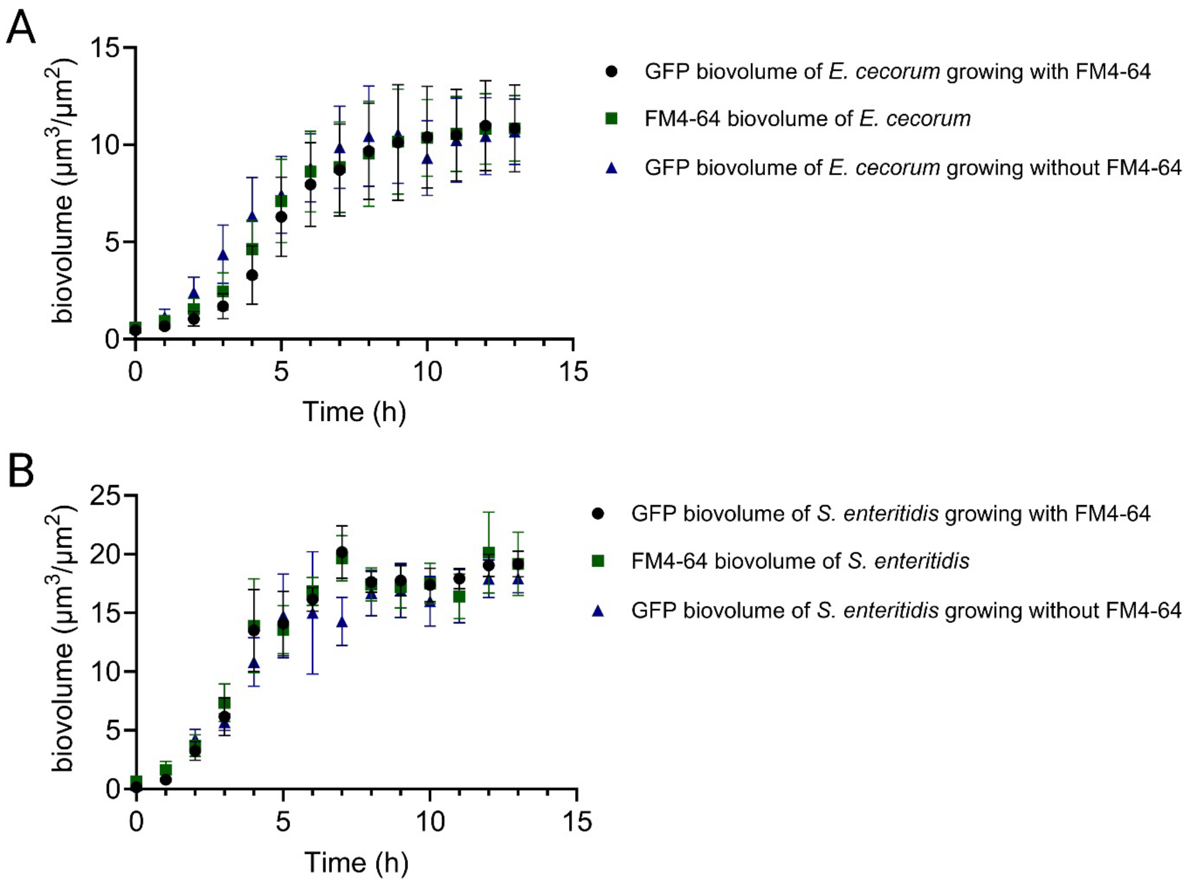

Supplementary Figure 16: Effect of the FM4-64 on pathogen growth. (A) Biovolumes of *E. cecorum* GFP biofilms grown with or without FM4-64 and (B) Biovolume of *S. enterica* GFP biofilms grown with and without FM4-64.

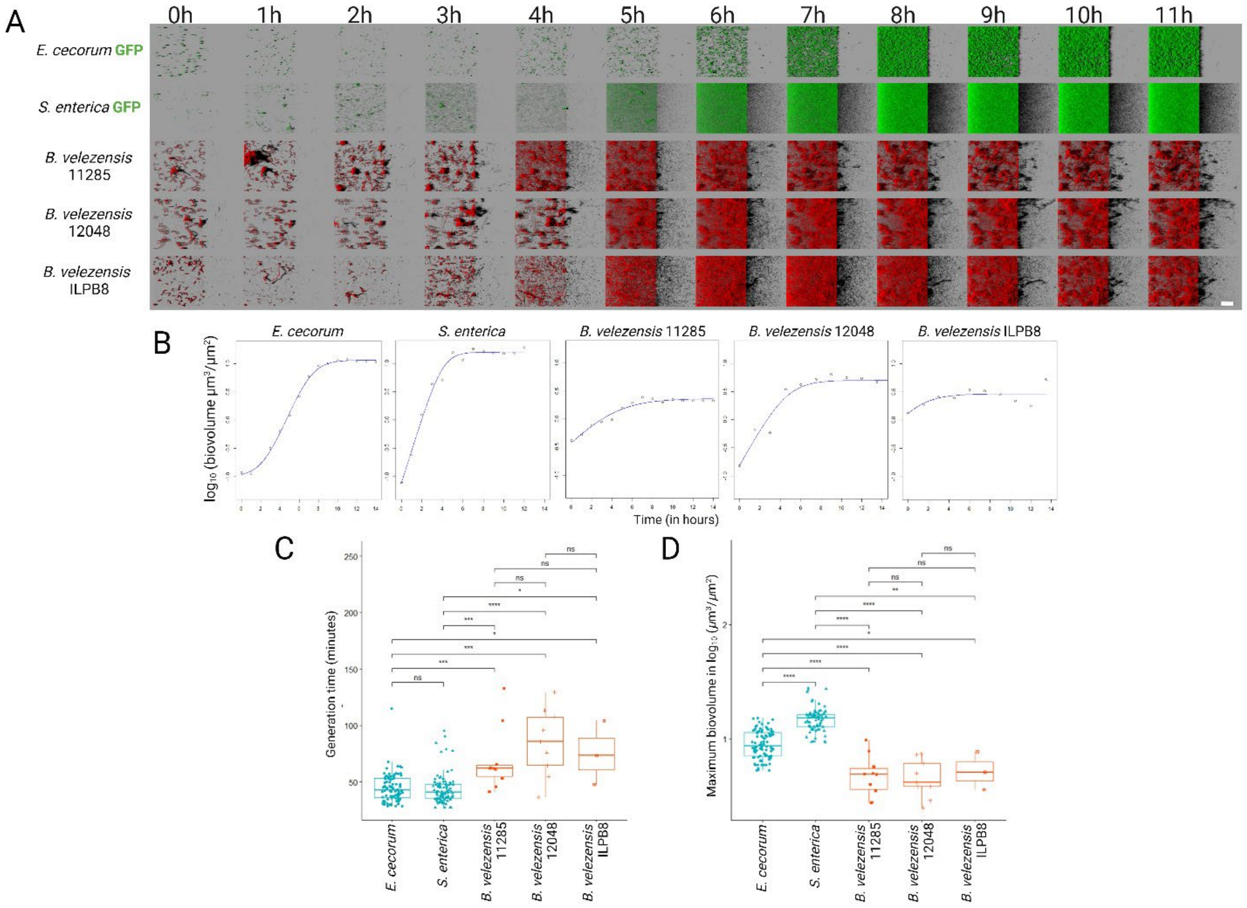

Supplementary Figure 17: Biofilm formation dynamics through CLSM kinetic studies. (A) Examples of CLSM visualisation of biofilm growth over time, with a representative example for each control condition. The green colour corresponds to GFP labelling, while the red colour represents FM4-64. The two-dimensional projections of biofilms were generated using IMARIS software in blend mode. Scale bar = 40 µm. (B) Illustration of biofilm growth modelling curves. The dots correspond to measured biovolumes, the line to the primary growth model fitted to observations. (C) Comparison of the generation times (D) and maximum biovolumes extracted using the mathematical model reached during monoculture experiments.

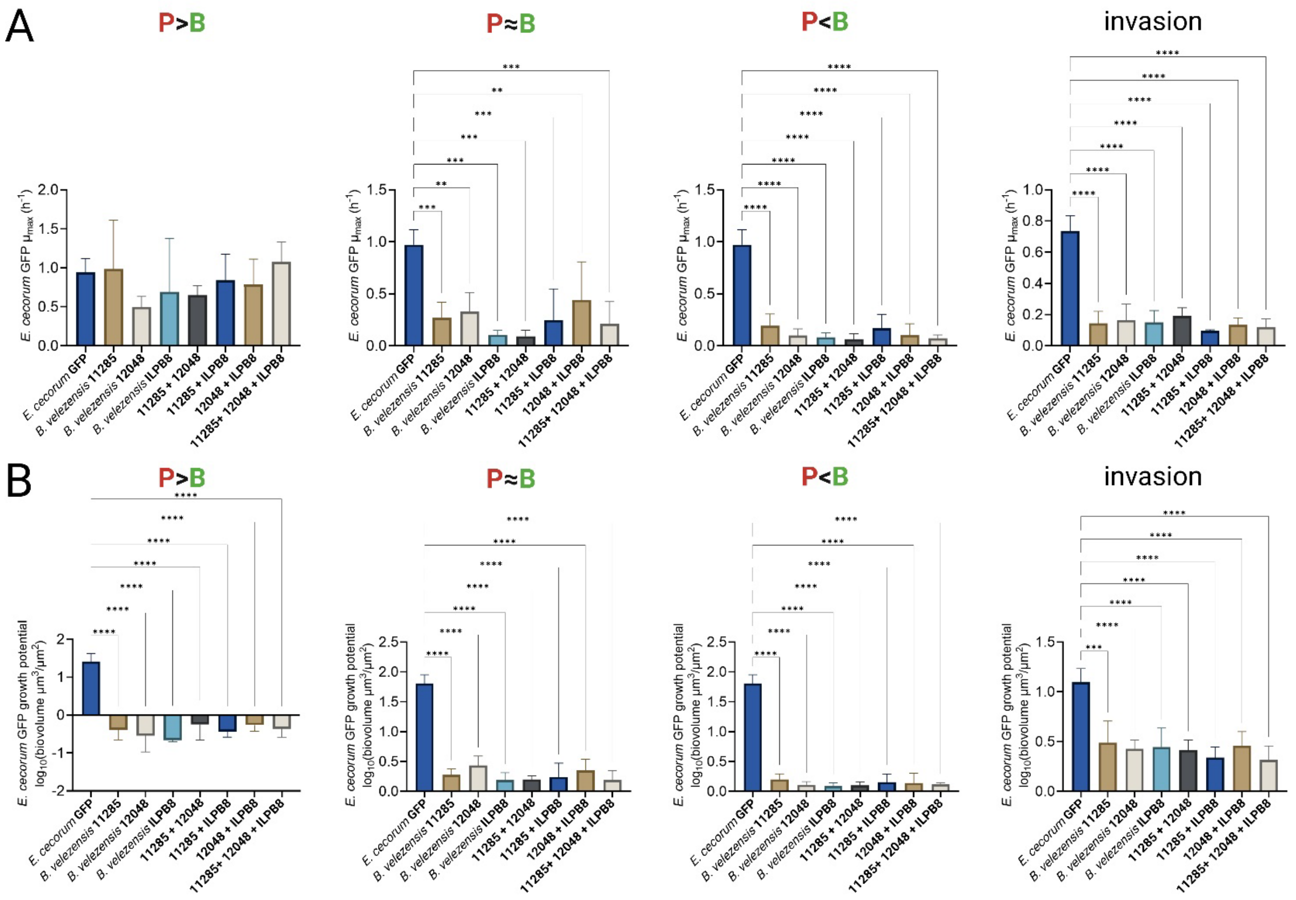

Supplementary Figure 18: Growth rates and growth potentials of *E. cecorum* in the presence of *B. velezensis* alone or in consortia. The initial biovolume ratios of *E. cecorum* GFP to *B. velezensis* were determined at the start of the experiment (ratio 1 = 1.4 (+/- 0.2), ratio 2 = 0.3 (+/- 0.06), ratio 3 = 0.03 (+/- 0.02), invasion = 0.2 (+/- 0.04)). (A) Correspond to the growth rate and (B) to the growth potential.

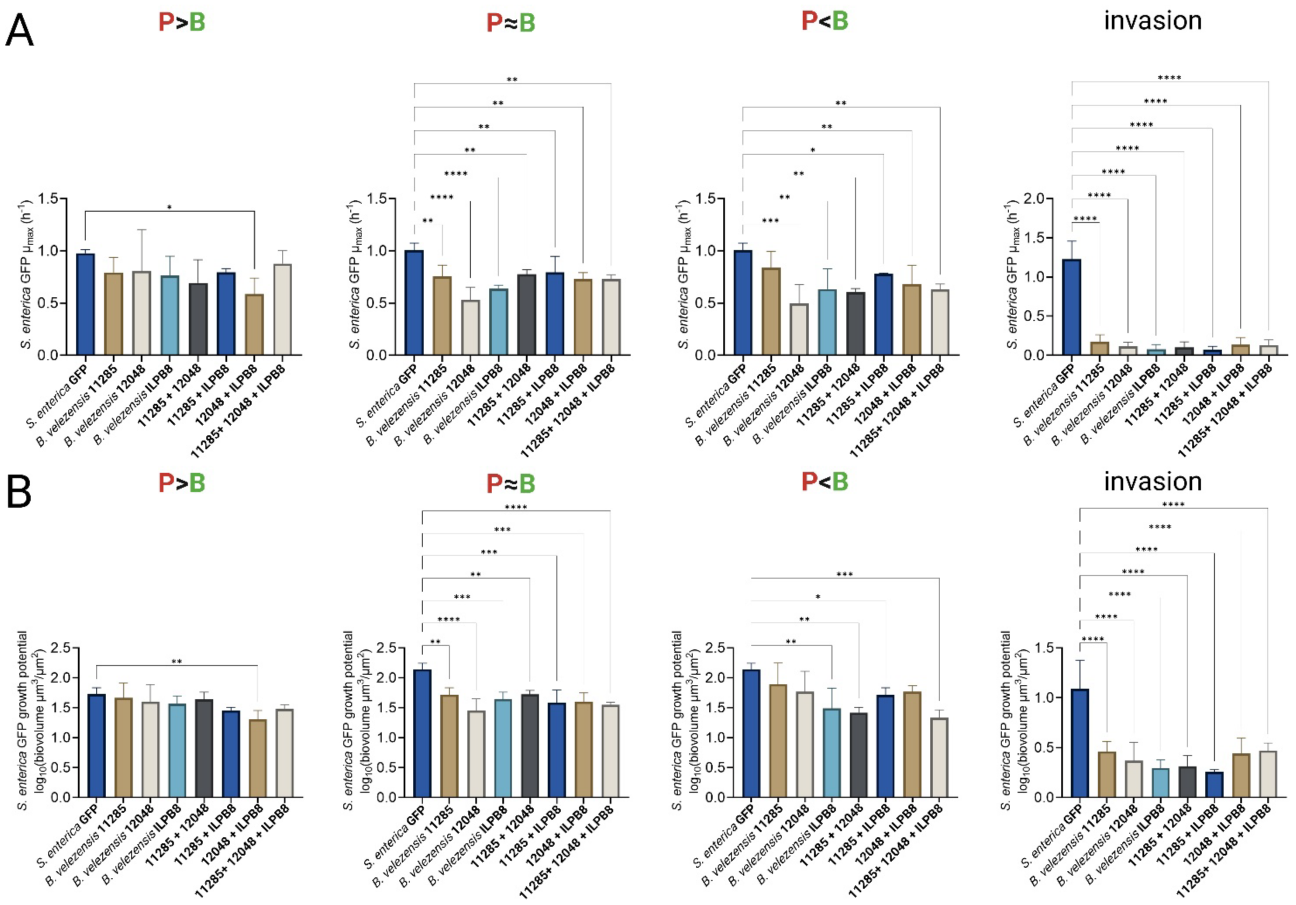

Supplementary Figure 19: Growth rates and growth potentials of *S. enterica* in the presence of *B. velezensis* alone or in consortia. The initial biovolume ratios of *S. enterica* GFP to *B. velezensis* were determined at the start of the experiment (ratio 1 = 3.2 (+/- 0.8), ratio 2 = 0.4 (+/- 0.1), ratio 3 = 0.1 (+/- 0.05), invasion = 4.8 (+/- 0.8)). (A) Correspond to the growth rate and (B) to the growth potential.

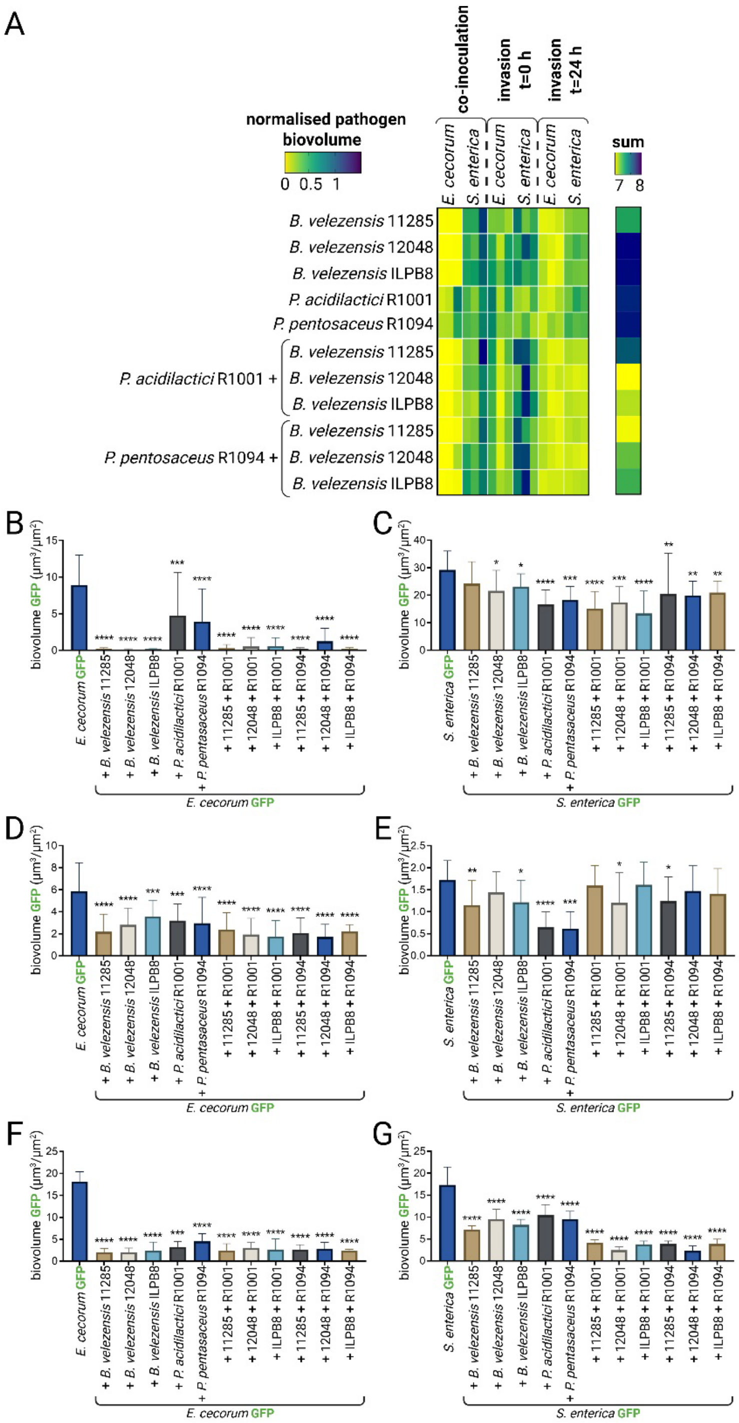

Supplementary Figure 20: Exclusion of *E. cecorum* and *S. enterica* by *B. velezensis*, *Pediococcus* spp. or their combinations in the co-incubation models. (A) A heatmap compiling all the results of GFP pathogen biovolumes in the presence of antagonistic strain candidates, normalised relative to the biovolumes of GFP pathogens alone. The detailed results are shown for (B) GFP-labelled *E. cecorum* in the co-inoculation model, (C) GFP-labelled *S. enterica* in the co-inoculation model, (D) GFP-labelled *E. cecorum* in the invasion t=0h model, (E) GFP-labelled *S. enterica* in the invasion t=0h model, (F) GFP-labelled *E. cecorum* in the invasion t=24h model, (G) GFP-labelled *S. enterica* in the invasion t=24h model. Error bars correspond to the standard deviation. The biovolume values of the GFP pathogen in the presence of the candidate antagonistic strains were compared with the biovolume of the pathogen alone.

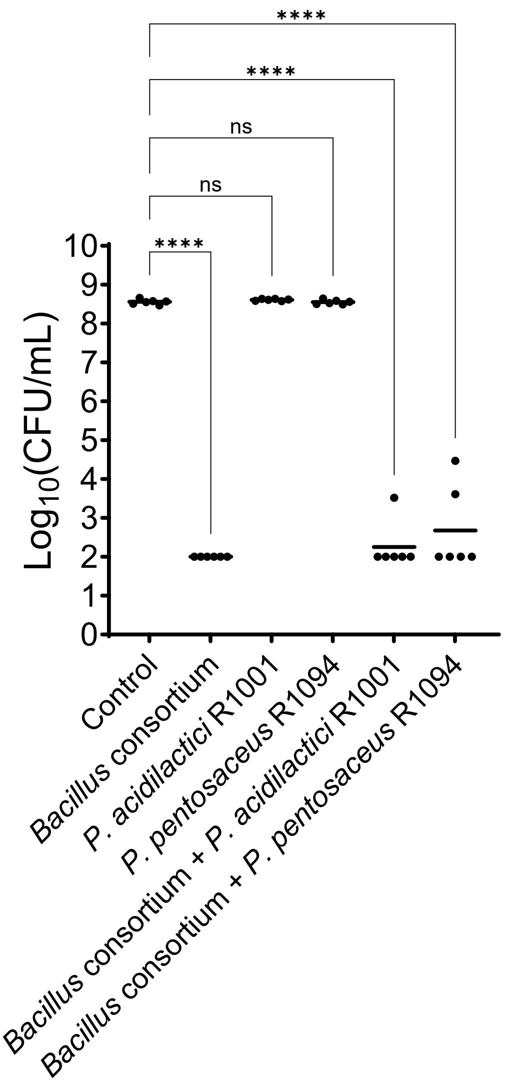

Supplementary Figure 21: Enumeration of *S. enterica* GFP on selective medium containing ampicillin, in the presence of ampicillin-sensitive competitor bacteria. The SynCom of *B. velezensis* strains 11285, 12048 and ILPB8 (*Bacillus* consortium) was co-cultured with *P. acidilactici* R1001 or with *P. pentosaceus* R1094 before the adhesion of *S. enterica* GFP in the invasion model for 11 hours. Six biological replicates were performed. The detection limit for this experiment is 2 log_{10} CFU/mL.

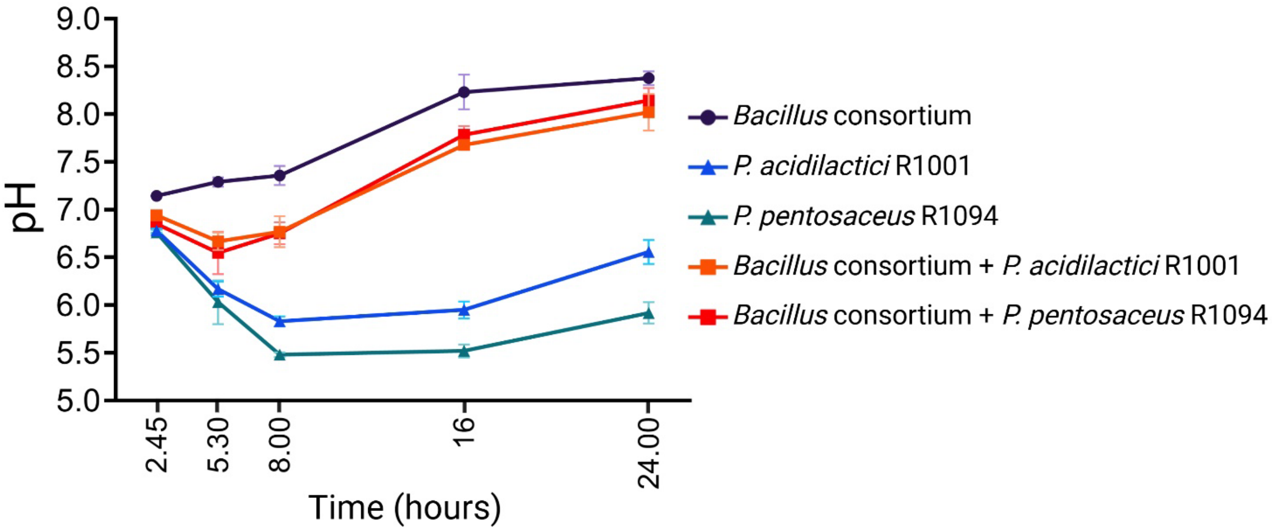

Supplementary Figure 22: Measurement of the pH of antagonistic strains alone or in consortia, in the invasion model. The invasion model was employed to monitor the pH of antagonistic pathogen-free biofilm solutions over time. The volume equivalent to 18 wells of a 96-well plate (200 µL each, totalling 3.6 mL) was measured using a pH metre (Mettler Toledo, FiveEasy F20 model, France) for each biological replicate. Each measurement for a biological replicate corresponds to one sacrificed 96-well plate. Initially, bacteria adhered to the well bottoms following the co-inoculation protocol, and measurements were taken at 24 hours. Subsequently, the invasion model was applied, where the medium was refreshed with fresh TS after co-inoculation, and measurements were conducted over time. Three biological replicates were performed for each measurement. The error bars represent standard errors.

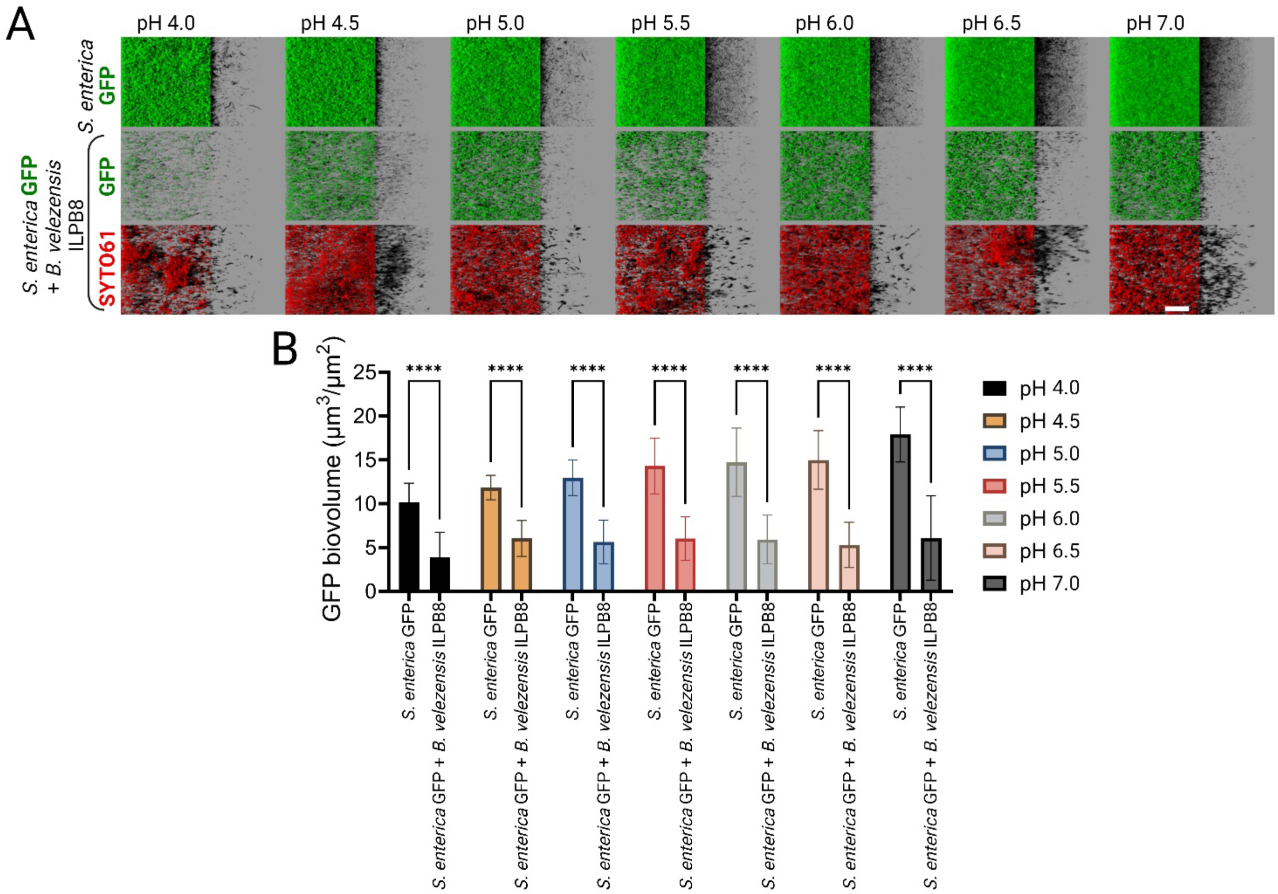

Supplementary Figure 23: Biofilm growth of *S. enterica* with *B. velezensis* ILPB8 at different acidic pH values. This experiment aims to investigate whether the pH reduction induced by *Pediococcus* spp. is involved in enhancing the exclusion of consortia composed of *Pediococcus* spp. and *B. velezensis* compared to *B. velezensis* alone, as observed in the invasion model. *S. enterica* GFP is cultured either alone or in the presence of *B. velezensis* ILPB8 in the invasion model at different acidic pH levels. HCl solution was incorporated to TSB medium to reach the desired value and then filtered with a 0.2 µm filter. *B. velezensis* biofilm is incubated for 24 hours, followed by the addition of *S. enterica* for a 1.5 h adhesion step before refreshing the media. (A) SYTO61 is added after 24 hours of incubation for observation using CLSM. The two-dimensional projections of biofilms were generated using IMARIS software in blend mode. Scale bar = 40 µm. (B) Through image analysis using the BiofilmQ software, the GFP biovolume of *S. enterica* alone is extracted and compared to the value obtained when *S. enterica* develops within a pre-established biofilm of *B. velezensis* ILPB8. The results demonstrate that medium acidification proportionally reduces the GFP biovolume of *S. enterica*. However, there is no significant difference in the *S. enterica* GFP biovolume in the presence of *B. velezensis* ILPB8 across different pH levels. Each value represents the average of three biological replicates, with four z-stack images each. The error bars correspond to standard errors.

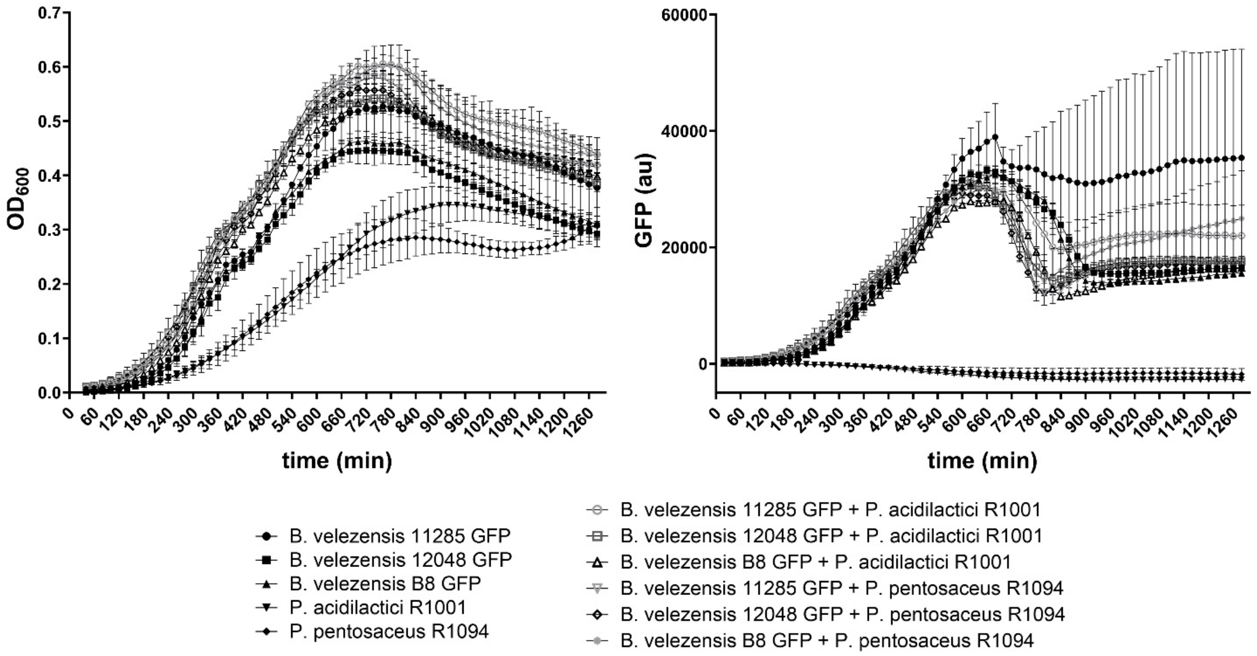

Supplementary Figure 24: Incompatibility of mixtures of *B. velezensis* and *Pediococcus* spp. in a planktonic lifestyle. The compatibility of *B. velezensis* expressing GFP and *Pediococcus* spp. Was investigated using the same protocol as the co-inoculation model. Following the adhesion step, ensuring the same initial biovolume for both bacterial partners, the cultures were resuspended. The cultures were continuously agitated to prevent bacterial biofilm formation. The OD600 was monitored over time using a Biotek plate reader (BioTek synergy h1, Agilent Technologies, USA) to follow the whole population. Fluorescence intensity between 500 and 550 was measured to assess the growth of *B. velezensis* GFP. Error bar represents SEM.

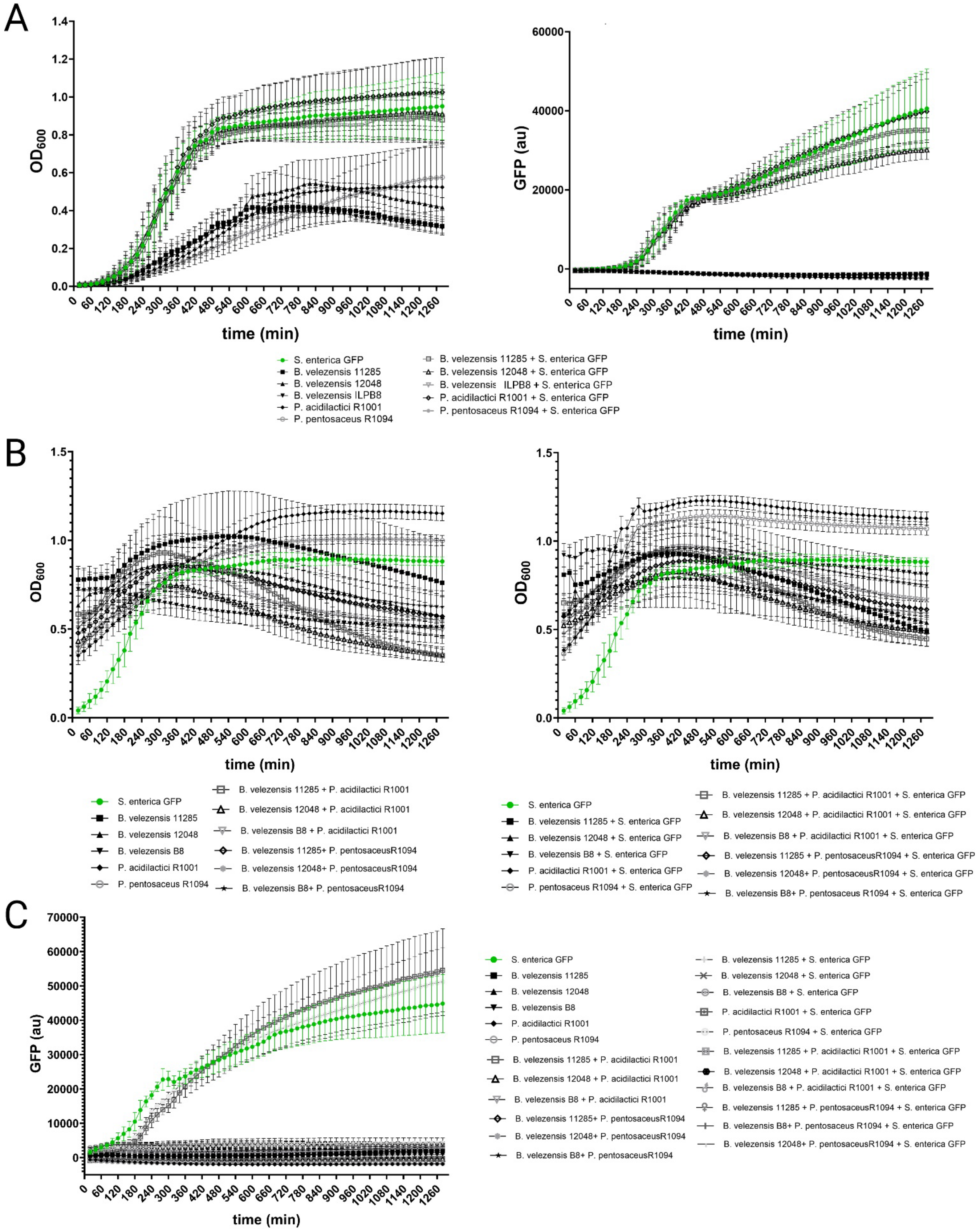
A
OD_{600}
time (min)
GFP (au)
time (min)
S. enterica GFP
B. velezensis 11285
B. velezensis 12048
B. velezensis ILPB8
P. acidilactici R1001
P. pentosaceus R1094
B. velezensis 11285 + S. enterica GFP
B. velezensis 12048 + S. enterica GFP
B. velezensis ILPB8 + S. enterica GFP
P. acidilactici R1001 + S. enterica GFP
P. pentosaceus R1094 + S. enterica GFP
B
OD_{600}
time (min)
OD_{600}
time (min)
S. enterica GFP
B. velezensis 11285
B. velezensis 12048
B. velezensis B8
P. acidilactici R1001
P. pentosaceus R1094
B. velezensis 11285 + P. acidilactici R1001
B. velezensis 12048 + P. acidilactici R1001
B. velezensis B8 + P. acidilactici R1001
B. velezensis 11285+ P. pentosaceusR1094
B. velezensis 12048+ P. pentosaceusR1094
B. velezensis B8+ P. pentosaceusR1094
S. enterica GFP
B. velezensis 11285 + S. enterica GFP
B. velezensis 12048 + S. enterica GFP
B. velezensis B8 + S. enterica GFP
P. acidilactici R1001 + S. enterica GFP
P. pentosaceus R1094 + S. enterica GFP
B. velezensis 11285 + P. acidilactici R1001 + S. enterica GFP
B. velezensis 12048 + P. acidilactici R1001 + S. enterica GFP
B. velezensis B8 + P. acidilactici R1001 + S. enterica GFP
B. velezensis 11285 + P. pentosaceusR1094 + S. enterica GFP
B. velezensis 12048+ P. pentosaceusR1094 + S. enterica GFP
B. velezensis B8+ P. pentosaceus R1094 + S. enterica GFP
C
GFP (au)
time (min)
S. enterica GFP
B. velezensis 11285
B. velezensis 12048
B. velezensis B8
P. acidilactici R1001
P. pentosaceus R1094
B. velezensis 11285 + P. acidilactici R1001
B. velezensis 12048 + P. acidilactici R1001
B. velezensis B8 + P. acidilactici R1001
B. velezensis 11285+ P. pentosaceusR1094
B. velezensis 12048+ P. pentosaceusR1094
B. velezensis B8+ P. pentosaceusR1094
B. velezensis 11285 + S. enterica GFP
B. velezensis 12048 + S. enterica GFP
B. velezensis B8 + S. enterica GFP
P. acidilactici R1001 + S. enterica GFP
P. pentosaceus R1094 + S. enterica GFP
B. velezensis 11285 + P. acidilactici R1001 + S. enterica GFP
B. velezensis 12048 + P. acidilactici R1001 + S. enterica GFP
B. velezensis B8 + P. acidilactici R1001 + S. enterica GFP
B. velezensis 11285 + P. pentosaceusR1094 + S. enterica GFP
B. velezensis B8+ P. pentosaceus R1094 + S. enterica GFP
B. velezensis 12048+ P. pentosaceusR1094 + S. enterica GFP

Supplementary Figure 25: Competition between *B. velezensis* or *Pediococcus* spp. and *S. enterica* in a planktonic lifestyle using different inoculation ratios. Following the adhesion step to validate the desired biovolume for both partners at the start of the experiment, the bacteria were resuspended and the cultures continuously agitated to prevent biofilm formation. The OD600 was monitored over time using a BioTek plate reader (Biotek Synergy H1, Agilent Technologies, USA) to track the entire population. Fluorescence intensity between 500 and 550 was measured to assess the growth of *S. enterica* GFP. The error bars represent the standard error of the mean (SEM). (A) The protocol used for the co-inoculation model was employed to investigate the antagonistic effect of *B. velezensis* and *Pediococcus* spp. on *S. enterica* expressing GFP. The left graph represents the measured OD600, and the right graph shows GFP intensity. (B) OD600 growth of individual strains is shown on the left graph or in the presence of *S. enterica* GFP on the right graph. (C) GFP intensity in the presence of antagonistic competitor strains. In this last graph, a growth of *S. enterica* GFP is only observed with *Pediococcus* spp. strains.

Supplementary references

1. Guéneau V, Rodiles A, Frayssinet B *et al.* Positive biofilms to control surface-associated microbial communities in a broiler chicken production system - a field study. *Front Microbiol* 2022;**13**:981747.

2. Malone CL, Boles BR, Lauderdale KJ *et al.* Fluorescent reporters for Staphylococcus aureus. *J Microbiol Methods* 2009;**77**:251–60.

3. Olson RD, Assaf R, Brettin T *et al.* Introducing the Bacterial and Viral Bioinformatics Resource Center (BV-BRC): a resource combining PATRIC, IRD and ViPR. *Nucleic Acids Res* 2023;**51**:D678–89.
